# Short-term associations between fine particulate air pollution and cardiovascular and respiratory mortality in 337 cities in Latin America

**DOI:** 10.1016/j.scitotenv.2024.171073

**Published:** 2024-04-10

**Authors:** Nelson Gouveia, Jordan L. Rodriguez-Hernandez, Josiah L. Kephart, Ana Ortigoza, Ricardo Morales Betancourt, Jose Luis Texcalac Sangrador, Daniel A. Rodriguez, Ana V. Diez Roux, Brisa Sanchez, Goro Yamada, The SALURBAL Group

**Affiliations:** a Department of Preventive Medicine, University of Sao Paulo Medical School, Sao Paulo, Brazil; b Urban Health Collaborative, Drexel Dornsife School of Public Health, Philadelphia, USA; c Department of Environmental and Occupational Health, Drexel Dornsife School of Public Health, Philadelphia, USA; d Department of Environmental and Social determinants for Health Equity, Pan American Health Organization, USA; e Department of Civil and Environmental Engineering, Universidad de Los Andes, Bogotá, Colombia; f Department of Environmental Health, Center for Population Health, National Institute of Public Health, Mexico; g Institute of Transportation Studies, University of California, Berkeley, CA, USA; h Department of City and Regional Planning and Institute Transportation Studies, University of California, Berkeley, USA; i Department of Epidemiology and Biostatistics, Drexel Dornsife School of Public Health, Philadelphia, USA

**Keywords:** Air pollution, Particulate matter, Cardiovascular mortality, Respiratory mortality

## Abstract

Ambient air pollution is a health concern in Latin America given its large urban population exposed to levels above recommended guidelines. Yet no studies have examined the mortality impact of air pollutants in the region across a wide range of cities. We assessed whether short-term levels of fine particulate matter (PM_2.5_) from modeled estimates, are associated with cardiovascular and respiratory mortality among adults in 337 cities from 9 Latin American countries. We compiled mortality, PM_2.5_ and temperature data for the period 2009–2015. For each city, we evaluated the association between monthly changes in PM_2.5_ and cardiovascular and respiratory mortality for sex and age subgroups using Poisson models, adjusted for seasonality, long-term trend, and temperature. To accommodate possibly different associations of mortality with PM_2.5_ by age, we included interaction terms between changes in PM_2.5_ and age in the models. We combined the city-specific estimates using a random effects meta-regression to obtain mortality relative risks for each sex and age group. We analyzed 3,026,861 and 1,222,623 cardiovascular and respiratory deaths, respectively, from a study population that represents 41 % of the total population of Latin America. We observed that a 10 μg/m^3^ increase in monthly PM_2.5_ is associated with an increase of 1.3 % (95 % confidence interval [CI], 0.4 to 2.2) in cardiovascular mortality and a 0.9 % increase (95 % CI -0.6 to 2.4) in respiratory mortality. Increases in mortality risk ranged between −0.5 % to 3.0 % across 6 sex-age groups, were larger in men, and demonstrated stronger associations with cardiovascular mortality as age increased. Socioeconomic, environmental and health contexts in Latin America are different than those present in higher income cities from which most evidence on air pollution impacts is drawn. Locally generated evidence constitutes a powerful instrument to engage civil society and help drive actions to mitigate and control ambient air pollution.

## Highlights

• Most evidence on health impacts of air pollution comes from high-income countries
• We examined whether PM_2.5_ is associated with cause specific mortality in adults of 337 cities in 9 Latin American countries
• A 10 μg/m^3^ increase in PM_2.5_ is associated with a 1.3% and a 0.9% increase in CVD and respiratory mortality respectively
• Increases in mortality were larger in men and associations with cardiovascular mortality were stronger at older ages
• Locally generated evidence can help drive actions to mitigate and control ambient air pollution in the region.

## Graphical abstract

**Figure:**
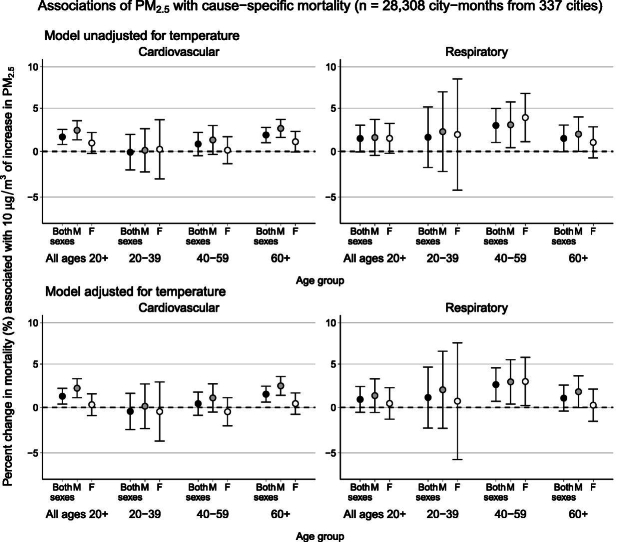

## 1 Introduction

Environmental pollution is one of the greatest risk factors for disease and premature mortality worldwide and to a large extent represents the unintended consequence of rapid and unplanned urbanization accompanied by motorization. Although the burden of disease attributable to some types of pollution such as indoor air pollution and water pollution has declined in recent decades, there has been an increase in deaths attributable to ambient levels of air pollution, especially in low- and middle-income countries (LMICs) (Fuller et al., 2022; Landrigan et al., 2018). In fact, ambient particulate matter pollution is among the risk exposures with the largest increases in disease burden from 2010 to 2019 (GBD 2019 Risk Factors Collaborators, 2020).

Recent assessments show that ambient air pollution is estimated to contribute to around 4.5 million premature deaths each year worldwide (Fuller et al., 2022). It affects individuals of all ages, although its impacts are particularly strong among the elderly (Gouveia and Fletcher, 2000), and may have differential effects by gender (Clougherty, 2010; Fuller et al., 2022).

A continuously growing body of evidence indicates that fine particulate air pollution (PM_2.5_) can harm health over a lifetime, affecting virtually every system in the human body. Numerous studies have shown the effects of PM_2.5_ on the lungs (Zhang et al., 2022), hearts (de Bont et al., 2022), brains and the central nervous system (Sîrbu et al., 2022; Sram et al., 2017), on pregnancy and birth-related outcomes and many other organs and systems (Markozannes et al., 2022; South Africa et al., 2019). The impact of exposure to PM_2.5_ on cardiovascular and respiratory diseases is of particular importance as these diseases are among the leading causes of death in most countries, especially for middle aged and older populations (GBD 2019 Diseases and Injuries Collaborators, 2020).

Nevertheless, most of the evidence of the impacts of PM_2.5_ on health comes from high-income countries in North America and Europe, and more recently from studies in China (Li et al., 2023). Estimates obtained in other parts of the world are necessary to understand these associations in different socioeconomic, environmental and health contexts. In addition, they are important to help policymakers outline the best strategies to mitigate the impacts of ambient air pollution on the health of their populations. Finally, regional or local studies may have greater power to generate engagement by local populations and decision-makers, since they can better perceive that the problem is affecting them and can result in solutions tailored to local conditions.

Latin America's high degree of urbanization and its rapid and unplanned growth have positioned urban air pollution as one of the most important environmental problems for the region. Exposure to PM_2.5_ is widespread in most urban centers, affecting all age groups but particularly children and the elderly (Gouveia et al., 2021). However, relatively few studies have examined the association between air pollutants and health effects in cities of this region. This is in part due to the sparsity of air quality data in the region, and the relatively late adoption of fine particulate matter (PM_2.5_) monitoring. The majority of the studies already carried out focused primarily on Brazil, Mexico and Chile, and to a lesser extent on Peru and Colombia. In all cases, they mostly involve a similar but limited number of large cities in each of these countries, which have air quality monitoring networks. Even a recent large study on particulate air pollution involving 652 cities around the world included just 14 cities from four Latin American countries in their analysis between PM_10_ and health outcomes, with that number decreasing to 7 cities in two countries when the analysis was done for PM_2.5_ (Liu et al., 2019).

In order to fill this important gap, the present study aims to assess whether documented levels of PM_2.5_ derived from satellite measurements at fine spatial resolution are associated with cardiovascular and respiratory mortality in adults in a large sample of Latin American cities.

## 2 Methods

### 2.1 Study setting

This study was conducted within the framework of the Salud Urbana en América Latina (SALURBAL) project, a multinational collaboration that has compiled and standardized data on demographics, social, health, and physical environments of 371 cities in 11 Latin American countries (Diez Roux et al., 2019). The SALURBAL cities encompass all urban agglomerations with populations of 100,000 or more in 2010 in the 11 participating countries. Each city is composed of adjacent administrative units (e.g., municipios, comunas, departamentos, depending on the country) (Quistberg et al., 2019). This analysis is based on 337 cities from nine countries for which mortality, population, PM_2.5_, and temperature data were available for the study period 2009–2015: Argentina, Brazil, Chile, Colombia, Costa Rica, El Salvador, Guatemala, Mexico, and Panama.

### 2.2 Data sources and data handling

We obtained individual mortality data from the vital registration systems of each country for individuals aged 20 years or older. The data included information on the place of residence, date of death, age, sex, and cause of death (in the form of ICD-10 codes) (Quistberg et al., 2019). Cardiovascular and respiratory mortality were identified using the World Health Organization (WHO) Global Health Estimate (GHE) 2015 classifications (ICD-10 codes for cardiovascular: I00-I99 and respiratory: J00-J06, J09-J18, J20-J22, J30-J98, H65-H66). Annual population data were obtained from national statistics offices (or other corresponding institutions), which included population projections stratified by city, year, age, and sex. We applied linear interpolation to obtain monthly population estimates from the annual mid-year population counts. To address the issue of under-registration of deaths, which varies by country and city, we applied a set of demographic methods called death distribution methods (Adair and Lopez, 2018; Hill et al., 2009; Hill, 2017; Peralta et al., 2019) to estimate the completeness of mortality registration by city, year, and sex category and adjusted the population counts accordingly (Bilal et al., 2021).

PM_2.5_ data, representing ground-level fine particulate matter, were obtained from the Atmospheric Composition Analysis Group at Washington University in St Louis. These data were generated using satellite measurements of aerosol optical depth and a chemical transport model and were calibrated to ground-based observations using geographically weighted regression (van Donkelaar et al., 2016). The data were presented as monthly mean concentrations (μg/m^3^) in a gridded format, with a spatial resolution of 1.1 km × 1.1 km. To estimate monthly city-level PM_2.5_ concentrations, we spatially weighted the values of PM_2.5_ based on the city's population in each grid cell to represent the exposure of the population more accurately and averaged the values for all grid cells within the city boundaries. For all countries we used 2010 estimates of the spatial distribution of the population from WorldPop (https://www.worldpop.org) to spatially weigh the city PM_2.5_ values except for Panama, where we used the Global Urban Footprint (https://www.un-spider.org/node/11424). This procedure gives proportionately greater weight to the air pollution concentrations where people live. This contrasts with taking the average concentration across the entire municipal boundary, which often includes inhabited and non-inhabited areas.

Temperature data were obtained from the land surface component of the 5th generation European ReAnalysis (ERA5-Land), produced by the European Centre for Medium-Range Weather Forecasts (ECMWF). The data is provided as gridded hourly temperature measurements with a native spatial resolution of 9 km × 9 km (Muñoz-Sabater et al., 2021), and contains estimates of air temperature at 2 m above the land surface (ambient temperature). However, ERA5-Land data omits pixels with >50 % water coverage, which affects with missing values a significant proportion of the SALURBAL cities (30 % of the total), some of which are located near the ocean. We corrected this issue through an imputation process (Hersbach et al., 2018) and then calculated monthly city-level averages by averaging the daily hourly temperatures and linking the raster gridded information to the city boundaries. As with the PM_2.5_ data, we derived a population-weighted measure of average monthly city temperature.

### 2.3 Statistical analysis

We assessed distributions of city-level cardiovascular and respiratory mortality rates and PM_2.5_ concentrations during the study period (2009–2015) using box plots and selected summary statistics (mean, standard deviation, and percentiles).

We performed exploratory data analyses to evaluate cross-sectional and longitudinal trends, check for seasonal patterns, and explore possible effect modification by age and sex of the association between PM_2.5_ levels and mortality (see Supplementary Material).

To estimate the adjusted relative change in cause-specific mortality associated with longitudinal changes in monthly PM_2.5_ overall and for each sex and age group, we employed a two-step regression that allowed us to account for seasonal mortality trends in each city.

First, we aggregated individual mortality data to construct monthly mortality counts. These counts were grouped by city, cause of death (cardiovascular or respiratory), sex (both sexes, males, or females), and age (all ages 20 years or above, or three age groups, 20–39, 40–59, and 60+ years). These datasets were linked to the corresponding city-level monthly PM_2.5_, annual population, and monthly temperature data. We then estimated the association between longitudinal changes in monthly PM_2.5_ and cause-specific mortality using a city-specific Poisson model adjusted for seasonal and long-term trends in mortality with a robust variance estimator to account for overdispersion in mortality, and used population counts as an offset. Temperature was modeled using a restricted cubic spline with knots at 10th, 75th, and 90th percentiles of city-specific distribution. We selected the number of knots and their locations based on the previous literature (Gasparrini et al., 2012, Gasparrini et al., 2015; Kephart et al., 2022) and chose the combination of knots that resulted in a better model fit visually.

We used a categorical variable corresponding to calendar month to adjust for seasonality and study month (1 to 84, 12 months for 7 years) to adjust for long-term trend. We fitted models for each sex (both sexes, male, and female) and for age-aggregated and age-stratified data separately. For the models using age-stratified counts, we included interaction terms between changes in PM_2.5_ and age. These terms allowed us to accommodate possibly different associations between changes in mortality and PM_2.5_ by age.

In the second step, we combined the city-specific estimates to determine the percentage change in mortality associated with a 10 μg/m^3^ increase in monthly PM_2.5_, for each age and sex group. To achieve this, we employed multivariate random effects meta-analysis (or equivalently), an intercept-only meta-regression model (Jackson et al., 2011). The use of multivariate meta regression allowed us to account for the dependence between age specific associations within the same city and to test whether the effect of PM_2.5_ on mortality differs across age groups. We used a 10 μg/m^3^ increment in PM_2.5_ to facilitate comparison of our results to with other studies, especially the ESCALA study (Romieu et al., 2012), the only multicity study previously conducted in Latin America.

Additionally, we examined if the association between changes in PM_2.5_ and mortality differed by the city's average PM_2.5_ over entire study period, by plotting estimated city-specific PM_2.5_-mortality associations against city-specific average PM_2.5_ (Figs. S5 and S6 of the supplement material). Since we did not find any sign of differences, we used the intercept-only model meta-regression described in the second step.

In order to examine if our results were influenced by temperature, as a sensitivity analysis, we repeat the modelling approach pulling out temperature.

## 3 Results

The number of cities examined in each country, the median size of city populations and the distribution of city populations by age groups are presented in Table 1. Brazil and Mexico contributed the largest number of study cities, while Central American countries contributed the fewest. Study cities have median populations of between 230,000 to 380,000 inhabitants and included several large cities such as São Paulo, Brazil, Mexico City, Mexico, Buenos Aires, Argentina, and San Jose, Costa Rica, among others. The 337 cities included in this analysis host 277 million inhabitants and represent approximately 41 % of the total population of Latin America. Individuals aged 20 to 39 represent about a third of the total population of cities, while the median percentage of older adults (60+) varies from 7.5 % to 14.8 % among countries. During the 84 months analyzed in this study there were 3,026,861 cardiovascular and 1,222,623 respiratory deaths observed. Ischemic heart disease and cerebrovascular disease accounted for nearly 62 % of CVD deaths, and lower respiratory infections and chronic obstructive pulmonary disease accounted for 74 % of respiratory deaths (supplement material Table S1).

**Table 1:** Characteristics of cities included in the analyses (median, 25th and 75th percentiles).

	Argentina	Brazil	Chile	Colombia	Costa Rica	El Salvador	Guatemala	Mexico	Panama
City level characteristics (median, 25th and 75th %tiles)									
Number of cities	28	152	20	35	1	3	3	92	3
City population in 100,000s^a^	2.9 (2.0,5.8)	2.4 (1.6,5.4)	2.4 (1.7,3.7)	3.4 (1.6,5.6)	25.1 (25.1,25.1)	2.7 (2.6,11.0)	2.8 (2.2,17.4)	3.8 (2.2,8.5)	2.3 (2.3,10.8)
% city pop 20–39^a^	31.6 (30.4,32.2)	33.6 (32.4,34.9)	31.0 (30.2,32.7)	31.6 (30.1,32.6)	35.0 (35.0,35.0)	32.9 (32.8,33.2)	34.5 (34.5,35.2)	32.6 (31.5,33.9)	30.5 (29.8,31.4)
% city pop 40–59^a^	21.4 (20.5,22.0)	25.1 (23.4,25.9)	25.3 (24.8,26.2)	23.8 (21.7,24.5)	24.2 (24.2,24.2)	20.5 (18.8,21.5)	17.4 (16.8,17.8)	21.7 (20.9,23.0)	23.0 (21.9,24.3)
% city pop 60+^a^	13.2 (11.9,15.9)	12.6 (10.3,14.4)	14.8 (13.6,15.5)	11.5 (10.0,13.7)	12.0 (12.0,12.0)	11.6 (10.9,11.7)	7.5 (7.4,7.7)	9.1 (8.2,10.2)	10.5 (9.6,11.6)

a Population statistics correspond to the year 2015.

Mortality rates for cardiovascular and respiratory diseases increased with age and were higher for those aged 60+ in both sexes and higher overall for males (Table 2 and supplement material Fig. S1). Mean levels of PM_2.5_ for the majority of cities and months examined were above 10 μg/m^3^. On average, the monthly mean level of PM_2.5_ across all cities was 16 μg/m^3^ and the maximum value observed was 126 μg/m^3^ (Table 2). The distribution of the mean levels of PM_2.5_ is highly skewed (supplement material Fig. S2).

**Table 2:** Monthly average PM_2.5_ and monthly mortality rates (*n* = 28,308 city-months from 337 cities).

	Age group	Mean	SD	Median	Min	25th percentile	75th percentile	Max
PM2.5^a^		16.0	7.7	14.6	2.2	10.8	19.2	126
CVD^b^	20–39	1.1	1.3	0.9	0.0	0.0	1.7	15.4
	40–59	9.8	5.5	9.0	0.0	6.1	12.6	65.8
	60+	104.8	31.4	101.1	0.0	84.8	120.8	471.1
Resp^b^	20–39	0.6	0.9	0.0	0.0	0.0	0.9	22.7
	40–59	2.6	2.6	2.2	0.0	0.0	3.7	33.9
	60+	42.3	20.5	39	0.0	28.4	52.7	191.4

a PM2.5 is expressed in μg/m^3^, included sea salt and dust, and was weighted by population.

b Cardiovascular and respiratory mortality is expressed in rates by 100,000 population.

When examining the crude association between changes in monthly PM_2.5_ and changes in mortality rates stratified by age and sex (supplement material Figs. S3 and S4), we observed that associations were stronger in the older age group, for both cardiovascular and respiratory mortality, but not much different by sex.

The adjusted percentage changes in cause-specific mortality associated with changes in PM_2.5_ are displayed in Table 3. Overall, we observed that a 10 μg/m^3^ increase in PM_2.5_ levels is associated with an increase of 1.3 % (95 % confidence interval [CI], 0.4 to 2.2) in cardiovascular mortality and an increase of 0.9 % (95 % CI -0.6 to 2.4) for respiratory mortality (which did not meet statistical significance). When stratifying by sex for all ages, the association with cardiovascular mortality was much stronger for males (increase of 2.2 % (95 % CI 1.1 to 3.3) than for females (0.3 %, 95 % CI -0.9 to 1.5). For respiratory mortality, we similarly observed a stronger association in males (increase of 1.3 %, 95 % CI -0.6 to 3.3) than for females (0.4 %, 95 % CI -1.3 to 2.2) but the magnitude of the difference between sexes was smaller and neither sex-stratified association achieved statistical significance.

**Table 3:** Estimated percent change in mortality (%) associated with 10 μg/m^3^ increase in PM_2.5_ by age group, 2009–2015 (n = 28,308 city-months from 337 cities).

	Both sexes		Males		Females	
	Change (95 % CI)	P-value	Change (95 % CI)	P-value	Change (95 % CI)	P-value
Cardiovascular						
All age groups	1.3 (0.4,2.2)	0.005	2.2 (1.1,3.3)	<0.001	0.3 (−0.9,1.5)	0.62
		0.002		<0.001		0.54
•20–39	−0.4 (−2.5,1.6)		0.1 (−2.3,2.7)		−0.5 (−3.7,2.9)	
•40–59	0.4 (−0.9,1.8)		1.1 (−0.5,2.7)		−0.5 (−2.0,1.1)	
•60+	1.5 (0.6,2.4)		2.5 (1.4,3.6)		0.4 (−0.8,1.6)	

Respiratory						
All age groups	0.9 (−0.6,2.4)	0.22	1.3 (−0.6,3.3)	0.17	0.4 (−1.3,2.2)	0.62
		0.06		0.13		0.20
•20–39	1.1 (−2.3,4.6)		2.0 (−2.3,6.5)		0.7 (−5.7,7.5)	
•40–59	2.6 (0.7,4.6)		2.9 (0.4,5.5)		3.0 (0.2,5.8)	
•60+	1.1 (−0.4,2.5)		1.8 (0.0,3.6)		0.3 (−1.5,2.1)	

Model included age (3 categories; 20–39, 40–64, 65+) + Interaction term between age and change in PM_2.5_ + Time (seasonality; 12 months as categorical) + Time (overall trend; calendar month) + Monthly temperature (restricted cubic spline with knots at 10th, 75th, and 90th percentiles of city-specific distribution).

Note: For age-stratified estimates, the global p-value comes from a 3 degrees of freedom test where the null hypothesis is that all age coefficients are equal to zero.

When stratifying by age, cardiovascular mortality associations were higher with increasing age in both sexes, with the strongest increase among males aged 60 years old or over (increase of 2.5 %, 95 % CI 1.4 to 3.6). For respiratory mortality, associations were strongest among middle-aged adults (aged 40–60) among both sexes but with much less precision. In summary, the percentage increases associated with a 10 μg/m^3^ increase in monthly PM_2.5_ ranged from −0.5 % to 3.0 % across 6 sex-age groups, were larger in men, and showed an increasing trend in the RR for cardiovascular mortality by age.

In the sensitivity analysis, models without adjustment for temperature provided larger estimates, especially for respiratory mortality, but maintaining the statistical significance (supplemental material Table S2).

We examined if the association between changes in PM_2.5_ and mortality differed by the city's average PM_2.5_, but we did not observe any sign of effect modification (supplemental material Figs. S5 and S6).

## 4 Discussion

This study revealed that increases in monthly levels of ambient PM_2.5_ are associated with increases in cardiovascular and respiratory mortality in adults in Latin American cities. Associations seem to be strongest for older male adults for deaths from cardiovascular disease.

Overall, the results found in this study are in line with multiple studies carried out in many different countries and also with the relatively few previous studies carried out in Latin America. An umbrella review of several meta-analyses published between 2010 and 2020 reports daily percent excess risk in cardiovascular mortality per 10 μg/m^3^ increase in PM_2.5_ ranging from 0.64 % (0.39; 0.97) to 1.00 % (1.00; 2.00) (de Bont et al., 2022). The large Multi-City Multi-Country (MCC) Study found increases of 0.55 % (95 % CI, 0.45 to 0.66) in daily cardiovascular mortality, and 0.74 % (95 % CI, 0.53 to 0.95) in daily respiratory mortality associated with the same 10 μg/m^3^ increase in PM_2.5_ concentrations. This multi-city study included 652 cities in all continents including 14 cities from Latin America (Liu et al., 2019). Similar results were found in China by Liang et al. (2018), with percent increases of 1.02 % (0.08 %, 1.97 %) and 0.09 % (−0.23 %, 0.42 %) for respiratory and cardiovascular mortality for an increase of 10 μg/m^3^ in short-term exposure to PM_2.5_, respectively.

The Multicity Study of Air Pollution and Mortality in Latin America (ESCALA), possibly the first of its kind conducted in the region, included 9 cities in 3 countries (Brazil, Chile and Mexico) and found an increase of 0.72 % (0.54 to 0.89) and 1.39 % (0.98 to 1.81) in cardiovascular and respiratory mortality in all ages for a 10 μg/m^3^ increase in daily levels of PM_10_ (Romieu et al., 2012). A meta-analysis of studies carried out in six cities in the same three countries showed a significant 2 % increased risk for respiratory mortality in all ages (RR = 1.02, 95 % CI, 1.02–1.02) associated with a 10 μg/m3 increase in PM_2.5_ levels (Fajersztajn et al., 2017). For cardiovascular mortality the increase in risk was 1 % (RR = 1.01, 95 % CI 1.01–1.02).

In general, the effect estimates we found are greater than those reported in other studies, including those conducted previously in Latin America. This is because these studies examined daily variations in pollution levels and mortality, that is, they evaluated acute or short-term effects. Our study used monthly data, since daily PM_2.5_ estimates derived from satellite readings were not available. Thus, we examined subacute effects, which are not necessarily of the same magnitude as acute effects. Monthly averages smooth and do not allow capturing the daily variability of pollution and its extreme values. Its use, presumably, would have less power to detect associations with acute health outcomes. In addition, studies using longer exposure windows have been associated with higher effects especially for respiratory mortality (Liang et al., 2018).

We also found that increases in mortality risk were slightly larger in the older population for cardiovascular diseases and for adults 40–60 years old for respiratory diseases. The fact that older adults seem to be more susceptible to the adverse effects of particulate matter air pollution has been evidenced in many studies, especially for cardiovascular diseases (de Bont et al., 2022; Romieu et al., 2012), and this is in part due to a larger burden of comorbidities among this population. In addition, we found more consistent associations for males. Sex differences in susceptibility to PM_2.5_ exposure have been reported previously. Fuller et al. (2022) analyzing the Global Burden of Disease data found that men are more likely to die from this exposure. Kuźma et al. (2020) found no sex differences in the impact of PM_2.5_ on all-cause mortality in Poland but in subgroup analysis they observed an effect of PM_2.5_ on cardiovascular deaths only in the male population. On the other hand, Liu et al. (2020) found that in China women were usually more vulnerable to ambient air pollution than men, and the exposure-response curves differed significantly between sexes. This differential susceptibility may be related to cultural or behavioral factors, such as men in general being more involved in outdoor activities and therefore more exposed to ambient air pollution, while women may be more subject to indoor air pollution. This could also be the result of biological or hormonal differences between men and women, or perhaps even a combination of both factors. In any case, a better understanding of gender differences can contribute to the design of more effective policies to mitigate the population's exposure to air pollution.

One of the strengths of this study is the large time period covered (7 years) and the temporal structure of the data which allowed investigating how longitudinal changes in pollution levels were related to variations in mortality, making a stronger case for the causal inference. Also, our analytical approach ensured an appropriate control for city-specific differences in seasonality and temporal trends, removing the challenges of time-invariant confounders by examining longitudinal changes within each individual city. But the major strength of this study is its wide geographical coverage. A large number of cities from 9 countries that account for a significant percentage of Latin America's adult population were included. This gives us greater confidence in the generalizability of the results to the region, with the reported associations being less prone to confounding factors or contextual differences inherent to the broad body of literature from the US, Europe, and China. This was possible because we used estimates of PM_2.5_ modeled from chemical transport models, land use, and satellite imagery, since air quality monitoring networks exist in only a few cities in the region. The use of satellite-based estimates for epidemiological studies is on the rise, as they allow the assessment of large population groups for which there was a lack of information on exposure. These measurements have been validated in studies that show a high correlation with ground-level PM_2.5_ (van Donkelaar et al., 2016) and are a useful tool for health research in areas such as Latin America where air monitor networks are sparse.

The present study also has some limitations. First, the data window used in our analysis was limited by the availability of temperature data for all cities. More recent data was not available at the time. Furthermore, only information on PM_2.5_ was available. Therefore, other important air pollutants present in urban areas (Kephart et al., 2023), which have documented health impacts, have not been examined. It is possible that these other pollutants may have partially contributed to the effects observed for PM_2.5_.

Over the past decades, many studies have examined the health effects of PM_2.5_. The mechanisms by which PM_2.5_ exerts its effects on the human body is already reasonably well established. These include sustained oxidative stress and systemic inflammation, and specifically for cardiovascular diseases, it also involves endothelial dysfunction, thrombotic pathways, and increased autonomic nervous system imbalance (Chen et al., 2022; de Bont et al., 2022). Currently, there is consistent evidence that PM_2.5_ not only aggravates the course of cardiovascular diseases, but also plays a role in their development (Konduracka and Rostoff, 2022).

In summary, our study found some evidence that current levels of ambient PM_2.5_ experienced by urban populations in Latin America are associated with mortality, especially mortality due to cardiovascular diseases in the elderly population. Considering the relatively high levels of air pollution in the region and the large number of individuals exposed to levels well above the WHO recommended guidelines for PM_2.5_ (Gouveia et al., 2021), the economic and social impacts are certainly important.

There is already a large body of evidence indicating that exposure to PM_2.5_ is associated with mortality, but the vast majority of these studies were conducted in large metropolitan areas of rich countries. Therefore, it is important to study the impacts of air pollution on cities in Latin America, a region with socioeconomic, environmental and health contexts that are quite different from the higher income cities where most of the evidence comes from. Many Latin American cities do not have public policies to control air pollution, and the population is largely unaware of the health risks resulting from this exposure. Thus, locally generated evidence constitutes a powerful instrument to engage civil society and decision-makers to drive actions to mitigate and control ambient air pollution.

## CRediT authorship contribution statement

**Nelson Gouveia:** Conceptualization, Data curation, Writing – original draft. **Jordan L. Rodriguez-Hernandez:** Data curation, Formal analysis, Writing – review & editing. **Josiah L. Kephart:** Validation, Writing – review & editing. **Ana Ortigoza:** Validation, Writing – review & editing. **Ricardo Morales Betancourt:** Validation, Writing – review & editing. **Jose Luis Texcalac Sangrador:** Validation, Writing – review & editing. **Daniel A. Rodriguez:** Validation, Writing – review & editing. **Ana V. Diez Roux:** Funding acquisition, Visualization, Writing – review & editing. **Brisa Sanchez:** Formal analysis, Methodology, Validation, Writing – review & editing. **Goro Yamada:** Conceptualization, Data curation, Formal analysis, Methodology, Validation, Writing – review & editing.

## Declaration of competing interest

The authors declare the following financial interests/personal relationships which may be considered as potential competing interests: Nelson Gouveia reports financial support was provided by Wellcome Trust. If there are other authors, they declare that they have no known competing financial interests or personal relationships that could have appeared to influence the work reported in this paper.

## Data Availability

Data will be made available on request.

## Appendix A Supplementary data

Supplementary material 1 Image 1

Supplementary material 2 Image 2

Supplementary material 3 Image 3
