# Supplementary material for: Short-term associations between fine particulate air pollution and cardiovascular and respiratory mortality in 337 cities in Latin America
Source: Sci Total Environ. 2024 Apr 10;920:171073. doi: 10.1016/j.scitotenv.2024.171073 (PMC10918459; doi:10.1016/j.scitotenv.2024.171073)
Supplement: Supplementary file 1 — Supplementary material 1 [file mmc1.docx]

Figure S1 – Monthly mortality rates by sex and age group during 2009-2015 (n= 28,308 city-months from 337 cities).


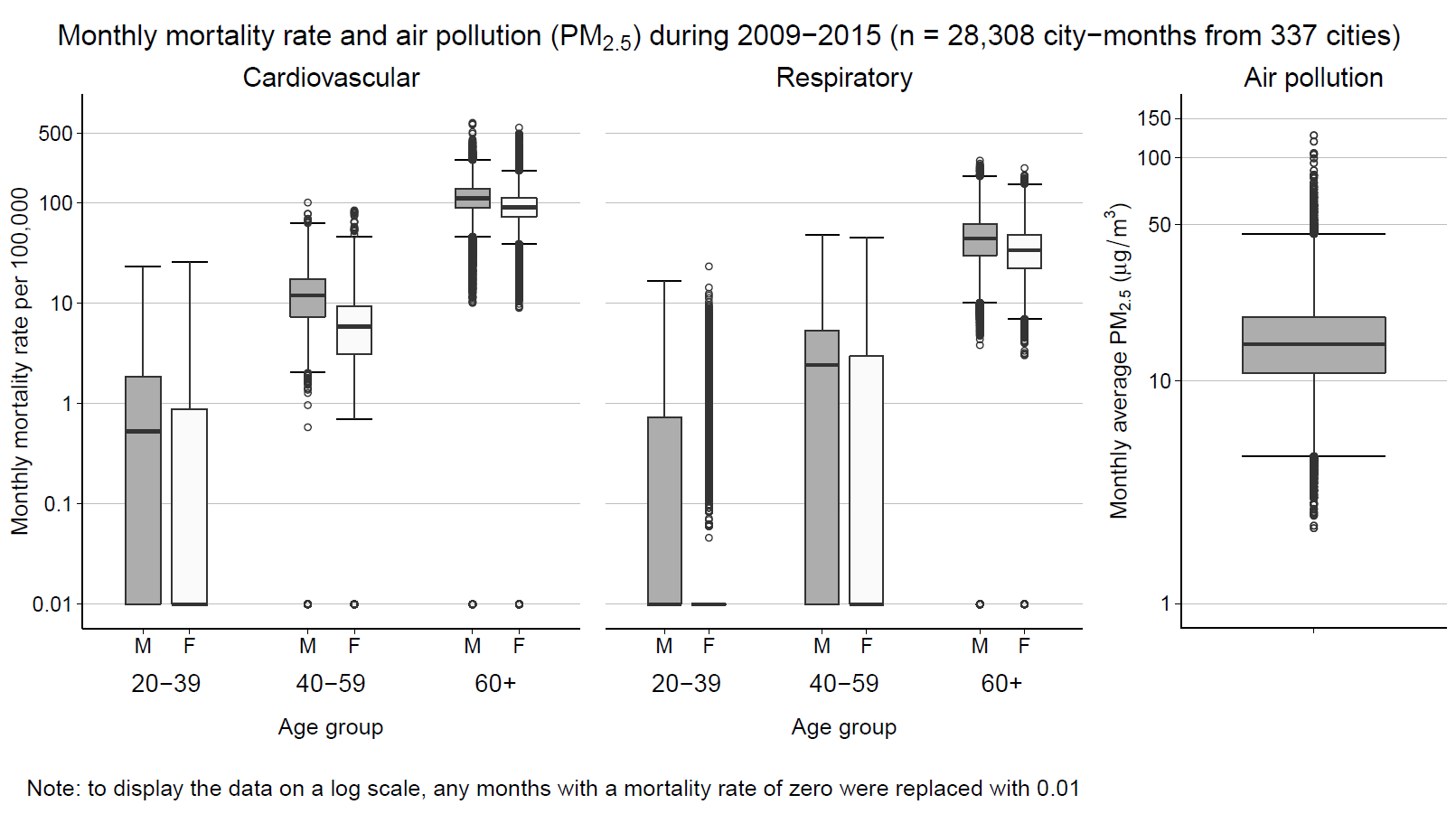

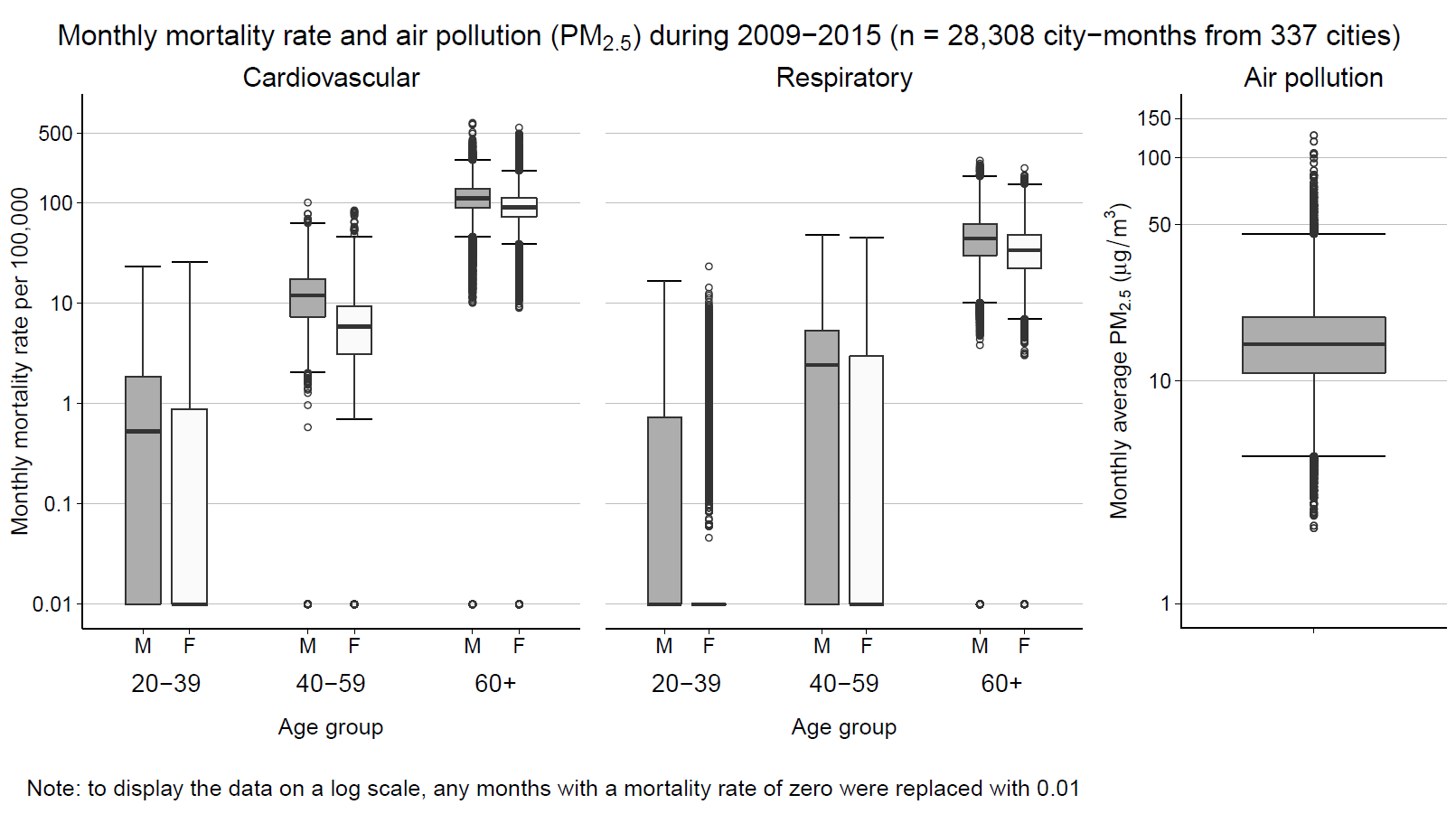


Figure S2 – Histogram of the monthly mean levels of PM2.5 during 2009-2015 (n= 28,308 city-months from 337 cities).


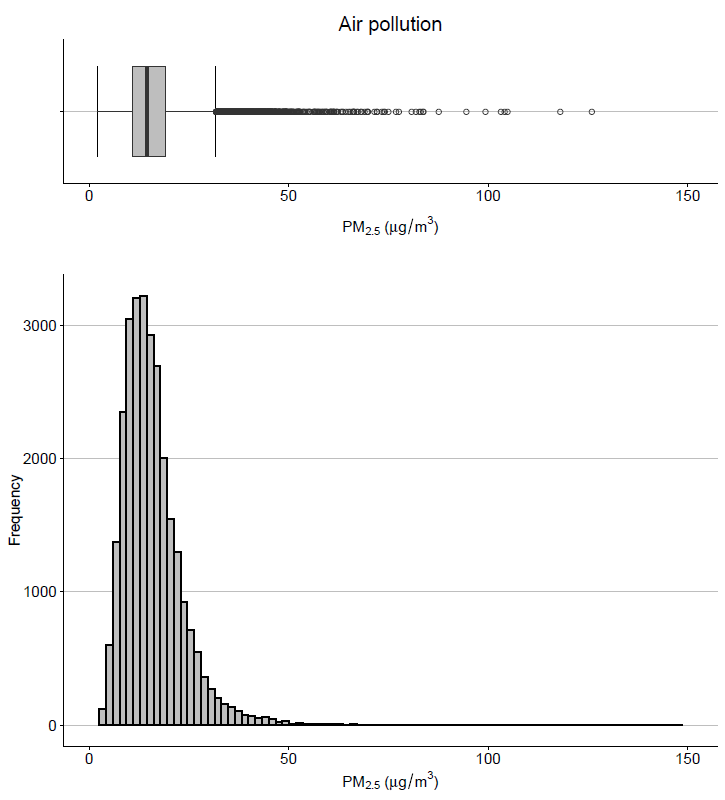


Table S1 - Specific diagnoses of cardiovascular and respiratory mortality from the Global Health Estimate (GHE) classification

| **GHE code** | **Cause** | **Number of deaths** | **%** |
| --- | --- | --- | --- |
| Cardiovascular | | | |
| 1080 | Cerebrovascular disease | 762,777 | 25.2 |
| 1060 | Hypertensive heart disease | 343,944 | 11.4 |
| 1090 | Inflammatory heart diseases | 109,395 | 3.6 |
| 1070 | Ischemic heart disease | 1,108,527 | 36.6 |
| 1100 | Other cardiovascular diseases | 685,616 | 22.6 |
| 1050 | Rheumatic heart disease | 16,602 | 0.6 |
| Total |  | 3,026,861 | 100.0 |
| Respiratory | | | |
| 1130 | Asthma | 18,038 | 1.5 |
| 1120 | Chronic obstructive pulmonary disease | 351,785 | 28.8 |
| 390 | Lower respiratory infections | 547,152 | 44.8 |
| 1140 | Other respiratory diseases | 303,762 | 24.8 |
| 410 | Otitis media | 672 | 0.1 |
| 400 | Upper respiratory infections | 1,214 | 0.1 |
| Total |  | 1,222,623 | 100.0 |

Figure S3 - Longitudinal associations (2009-2015) of cardiovascular mortality vs PM_2.5_ by sex and age group at city level (n= 28,308 city-months from 337 cities)


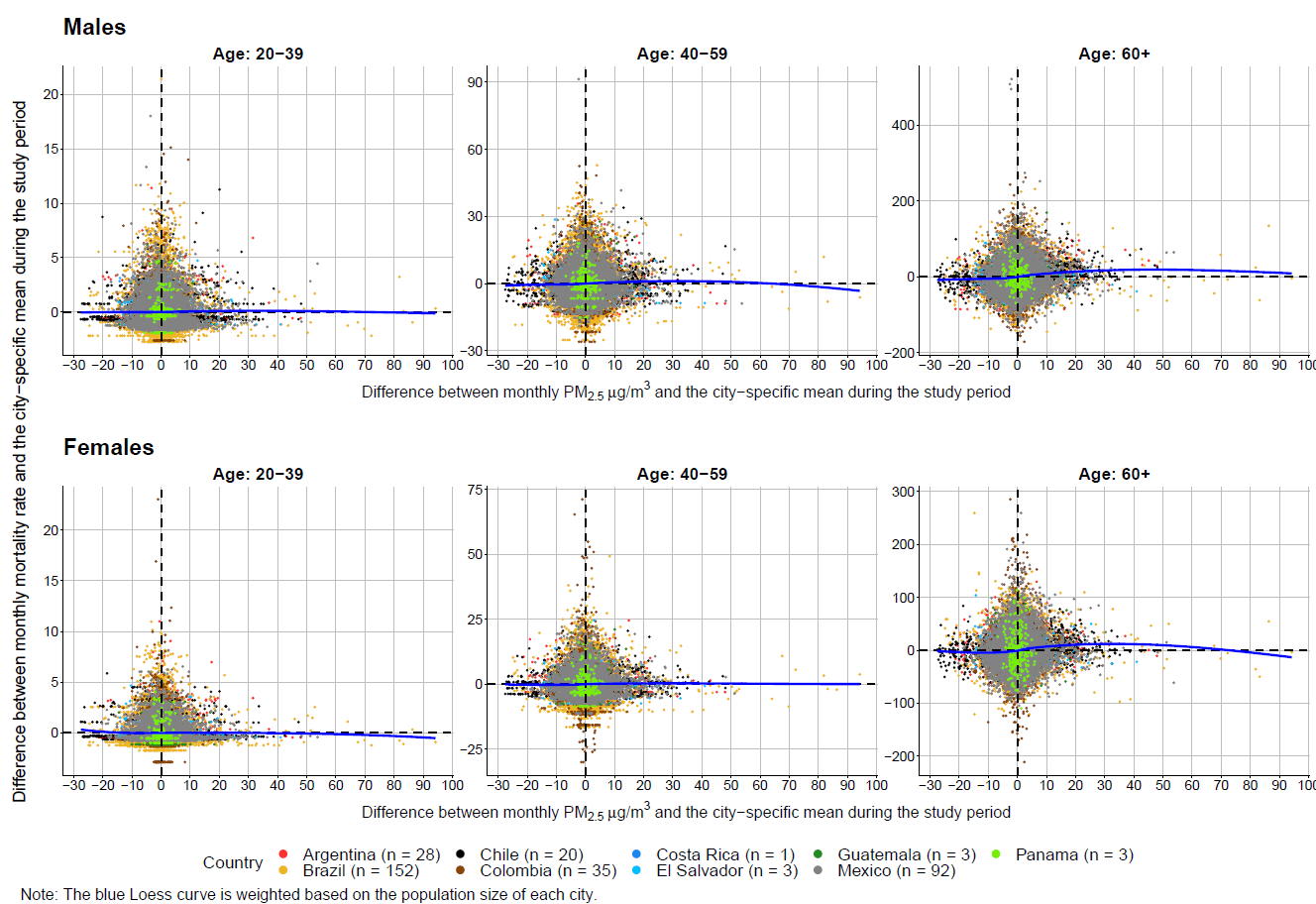


Figure S4 - Longitudinal associations (2009-2015) of respiratory mortality vs PM_2.5_ by sex and age group at city level (n= 28,308 city-months from 337 cities)


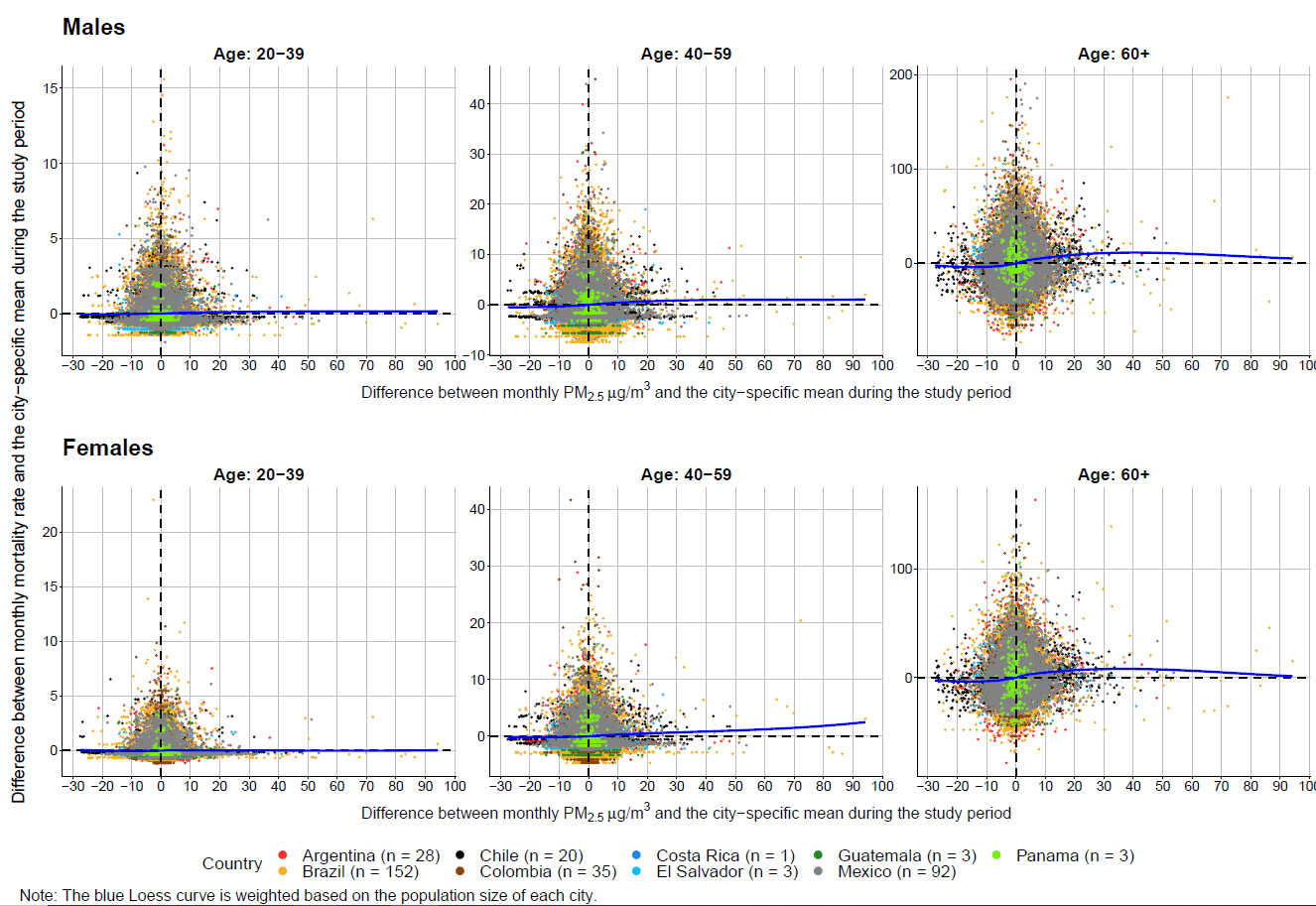


Figure S5 – associations between changes in PM2.5 and cardiovascular mortality by the city’s average PM_2.5_ (n= 337 cities)


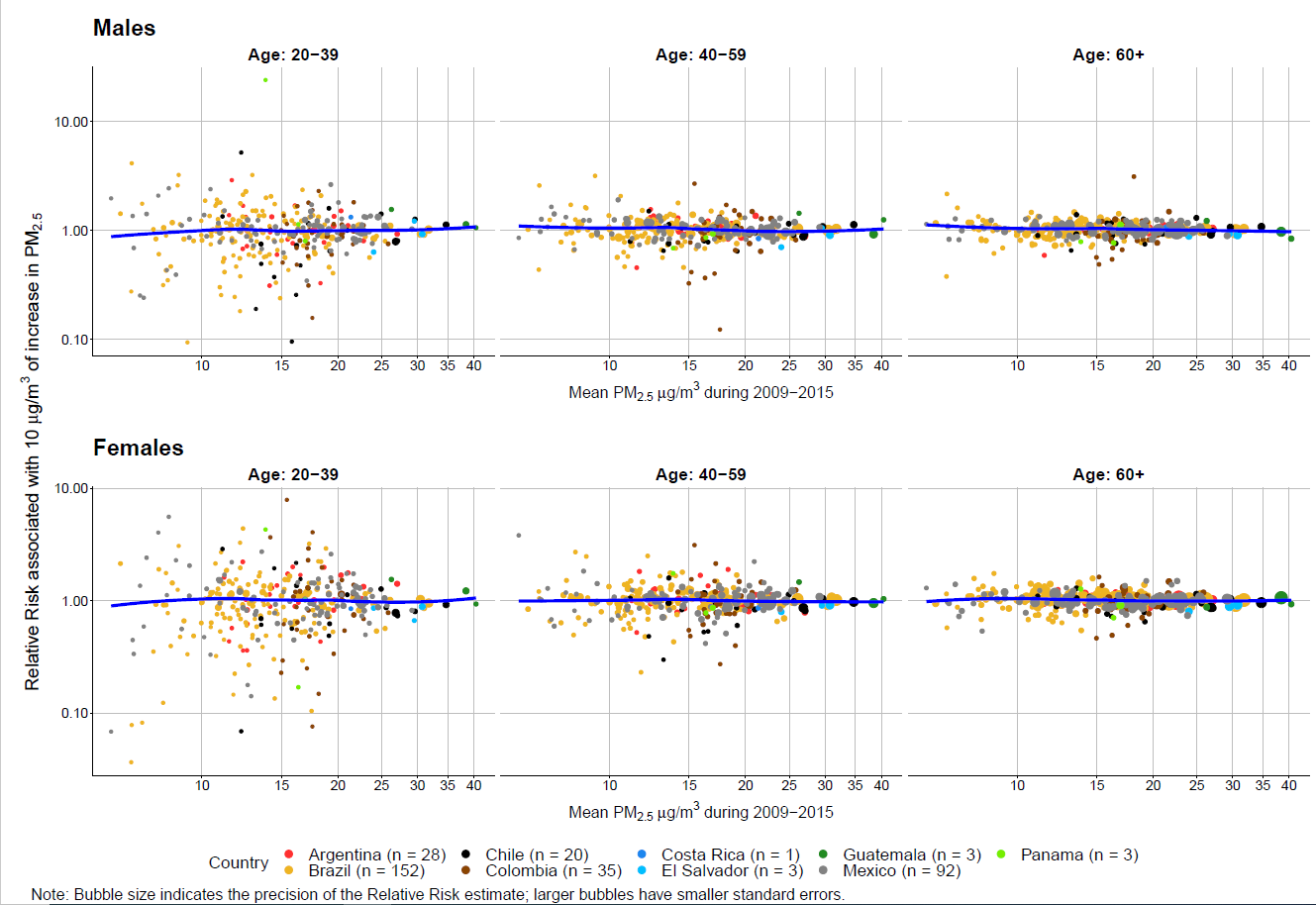


Figure S6 - associations between changes in PM2.5 and respiratory mortality by the city’s average PM_2.5_ (n= 337 cities)


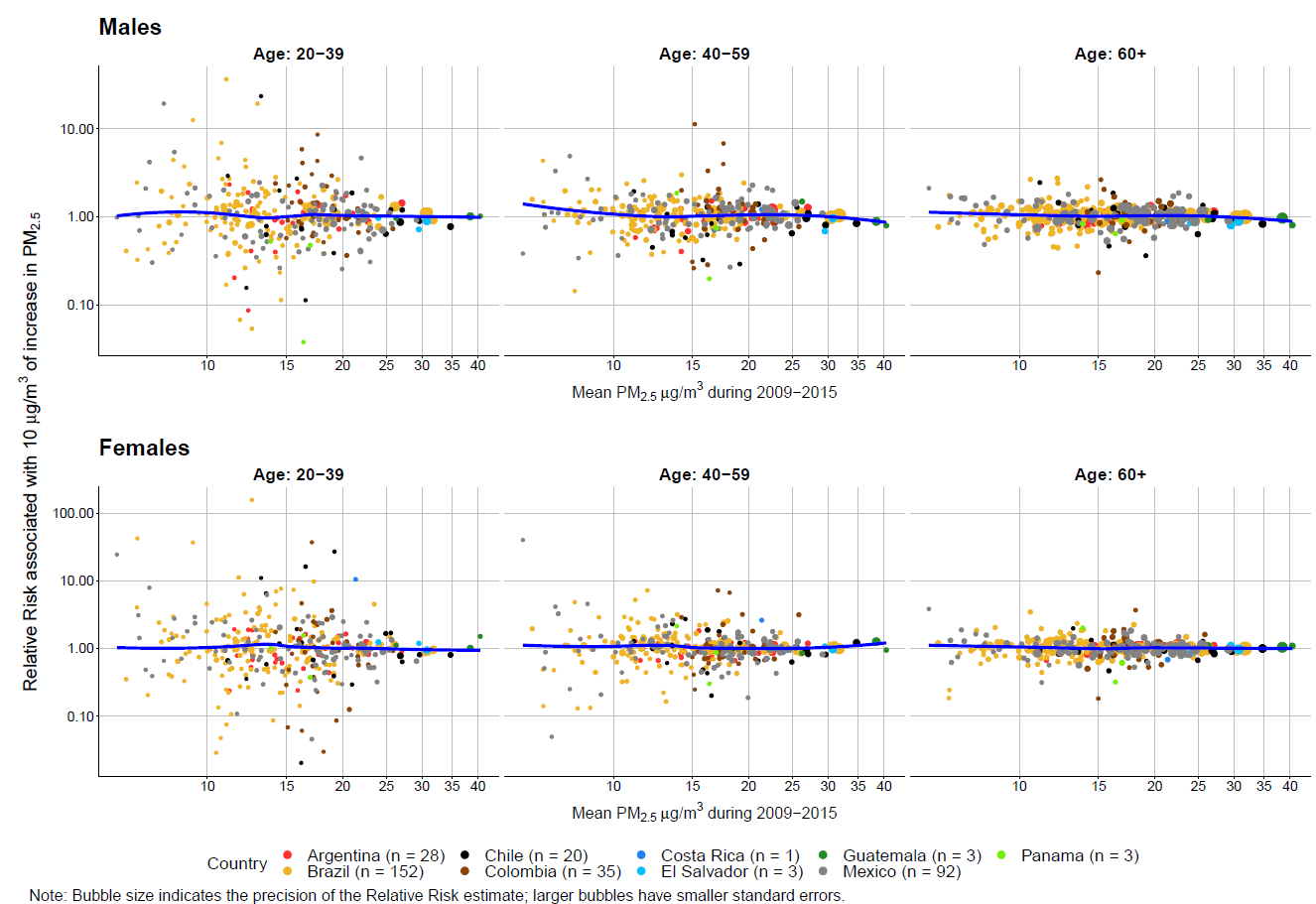


Table S2.

Percent change in mortality (%) associated with 10μg/m^3^ of increase in PM_2.5_ by age group, 2009-2015 (n = 28,308 city-months from 337 cities)

|  | **Both sexes** | | **Males** | | **Females** | |
| --- | --- | --- | --- | --- | --- | --- |
|  | **Change (95% CI)** | **P-value** | **Change (95% CI)** | **P-value** | **Change (95% CI)** | **P-value** |
| **Cardiovascular:** | | | | | | |
| All age groups | **1.6 ( 0.8,2.5)** | **<.001** | **2.4 ( 1.3,3.5)** | **<.001** | 1.0 (-0.2,2.2) | 0.11 |
|  |  | **<.001** |  | **<.001** |  | 0.22 |
| •20-40 | -0.1 (-2.1,1.9) |  | 0.1 (-2.3,2.6) |  | 0.2 (-3.0,3.6) |  |
| •40-60 | 0.8 (-0.5,2.2) |  | 1.3 (-0.3,3.0) |  | 0.1 (-1.4,1.7) |  |
| •60+ | **1.9 ( 1.0,2.7)** |  | **2.6 ( 1.6,3.7)** |  | 1.1 (-0.1,2.3) |  |
| **Respiratory:** | | | | | | |
| All age groups | 1.5 ( 0.0,3.0) | 0.06 | 1.6 (-0.5,3.7) | 0.13 | 1.5 (-0.2,3.2) | 0.09 |
|  |  | **0.03** |  | 0.12 |  | **0.05** |
| •20-40 | 1.6 (-1.8,5.1) |  | 2.3 (-2.2,6.9) |  | 1.9 (-4.3,8.5) |  |
| •40-60 | **3.0 ( 1.0,5.0)** |  | **3.0 ( 0.4,5.7)** |  | **3.9 ( 1.1,6.7)** |  |
| •60+ | 1.5 ( 0.0,3.0) |  | 2.0 ( 0.0,4.0) |  | 1.0 (-0.7,2.8) |  |

Estimates from a model which included age (3 categories; 20-39, 40-64, 65+) + Interaction term between age and change in PM_2.5_ + Time (seasonality; 12 months as categorical) + Time (overall trend; calendar month)

Note: For age-stratified estimates, the global p-value comes from a 3 degrees of freedom test where the null hypothesis is that all age coefficients are equal to zero
