# Supplementary material for: Short-term associations between fine particulate air pollution and cardiovascular and respiratory mortality in 337 cities in Latin America
Source: Sci Total Environ. 2024 Apr 10;920:171073. doi: 10.1016/j.scitotenv.2024.171073 (PMC10918459; doi:10.1016/j.scitotenv.2024.171073)

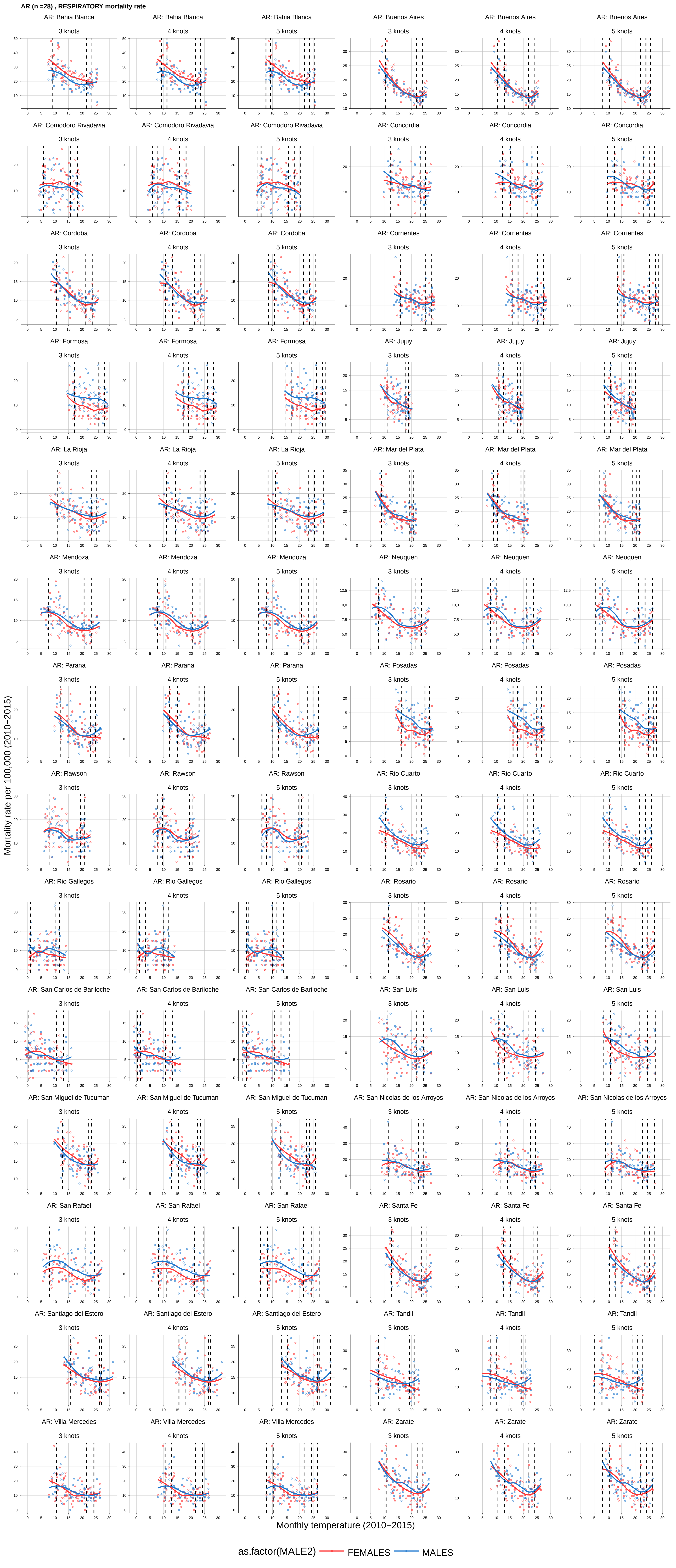
AR (n =28) , RESPIRATORY mortality rate
AR: Bahia Blanca
3 knots
4 knots
5 knots
AR: Buenos Aires
AR: Comodoro Rivadavia
AR: Concordia
AR: Cordoba
AR: Corrientes
AR: Formosa
AR: Jujuy
AR: La Rioja
AR: Mar del Plata
AR: Mendoza
AR: Neuquen
AR: Parana
AR: Posadas
AR: Rawson
AR: Rio Cuarto
AR: Rio Gallegos
AR: Rosario
AR: San Carlos de Bariloche
AR: San Luis
AR: San Miguel de Tucuman
AR: San Nicolas de los Arroyos
AR: San Rafael
AR: Santa Fe
AR: Santiago del Estero
AR: Tandil
AR: Villa Mercedes
AR: Zarate
Mortality rate per 100,000 (2010−2015)
Monthly temperature (2010−2015)
as.factor(MALE2)
FEMALES
MALES

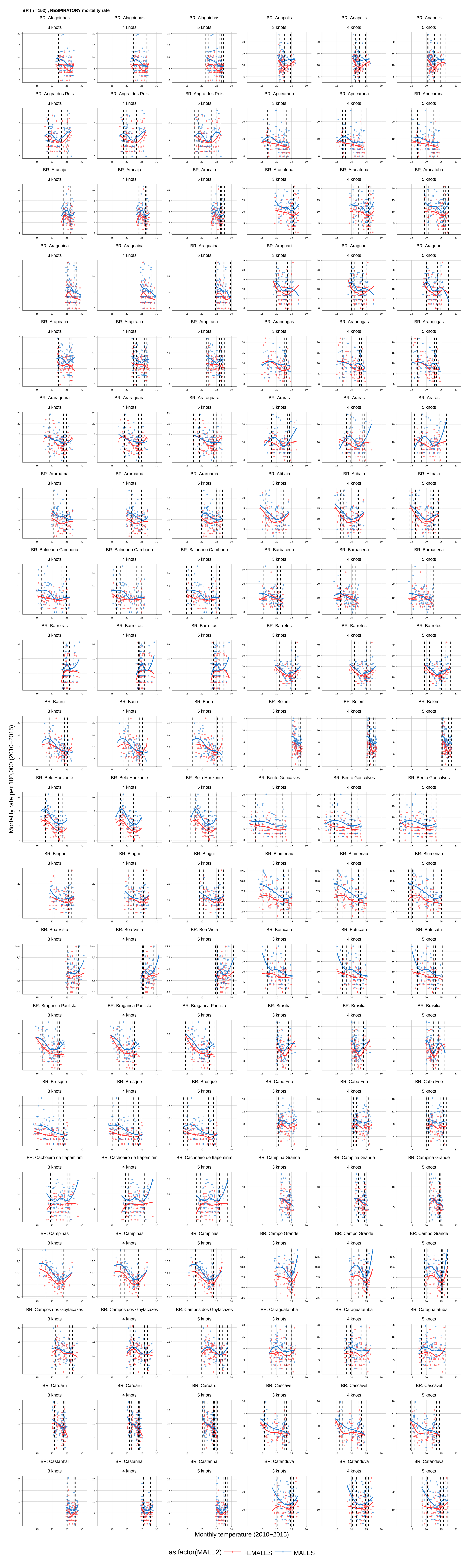
BR (n =152) , RESPIRATORY mortality rate
BR: Alagoinhas 3 knots | BR: Alagoinhas 4 knots | BR: Alagoinhas 5 knots | BR: Anapolis 3 knots | BR: Anapolis 4 knots | BR: Anapolis 5 knots
BR: Angra dos Reis 3 knots | BR: Angra dos Reis 4 knots | BR: Angra dos Reis 5 knots | BR: Apucarana 3 knots | BR: Apucarana 4 knots | BR: Apucarana 5 knots
BR: Aracaju 3 knots | BR: Aracaju 4 knots | BR: Aracaju 5 knots | BR: Aracatuba 3 knots | BR: Aracatuba 4 knots | BR: Aracatuba 5 knots
BR: Araguaina 3 knots | BR: Araguaina 4 knots | BR: Araguaina 5 knots | BR: Araguari 3 knots | BR: Araguari 4 knots | BR: Araguari 5 knots
BR: Arapiraca 3 knots | BR: Arapiraca 4 knots | BR: Arapiraca 5 knots | BR: Arapongas 3 knots | BR: Arapongas 4 knots | BR: Arapongas 5 knots
BR: Araraquara 3 knots | BR: Araraquara 4 knots | BR: Araraquara 5 knots | BR: Araras 3 knots | BR: Araras 4 knots | BR: Araras 5 knots
BR: Araruama 3 knots | BR: Araruama 4 knots | BR: Araruama 5 knots | BR: Atibaia 3 knots | BR: Atibaia 4 knots | BR: Atibaia 5 knots
BR: Balneario Camboriu 3 knots | BR: Balneario Camboriu 4 knots | BR: Balneario Camboriu 5 knots | BR: Barbacena 3 knots | BR: Barbacena 4 knots | BR: Barbacena 5 knots
BR: Barreiras 3 knots | BR: Barreiras 4 knots | BR: Barreiras 5 knots | BR: Barretos 3 knots | BR: Barretos 4 knots | BR: Barretos 5 knots
BR: Bauru 3 knots | BR: Bauru 4 knots | BR: Bauru 5 knots | BR: Belem 3 knots | BR: Belem 4 knots | BR: Belem 5 knots
BR: Belo Horizonte 3 knots | BR: Belo Horizonte 4 knots | BR: Belo Horizonte 5 knots | BR: Bento Goncalves 3 knots | BR: Bento Goncalves 4 knots | BR: Bento Goncalves 5 knots
BR: Birigui 3 knots | BR: Birigui 4 knots | BR: Birigui 5 knots | BR: Blumenau 3 knots | BR: Blumenau 4 knots | BR: Blumenau 5 knots
BR: Boa Vista 3 knots | BR: Boa Vista 4 knots | BR: Boa Vista 5 knots | BR: Botucatu 3 knots | BR: Botucatu 4 knots | BR: Botucatu 5 knots
BR: Braganca Paulista 3 knots | BR: Braganca Paulista 4 knots | BR: Braganca Paulista 5 knots | BR: Brasilia 3 knots | BR: Brasilia 4 knots | BR: Brasilia 5 knots
BR: Brusque 3 knots | BR: Brusque 4 knots | BR: Brusque 5 knots | BR: Cabo Frio 3 knots | BR: Cabo Frio 4 knots | BR: Cabo Frio 5 knots
BR: Cachoeiro de Itapemirim 3 knots | BR: Cachoeiro de Itapemirim 4 knots | BR: Cachoeiro de Itapemirim 5 knots | BR: Campina Grande 3 knots | BR: Campina Grande 4 knots | BR: Campina Grande 5 knots
BR: Campinas 3 knots | BR: Campinas 4 knots | BR: Campinas 5 knots | BR: Campo Grande 3 knots | BR: Campo Grande 4 knots | BR: Campo Grande 5 knots
BR: Campos dos Goytacazes 3 knots | BR: Campos dos Goytacazes 4 knots | BR: Campos dos Goytacazes 5 knots | BR: Caraguatatuba 3 knots | BR: Caraguatatuba 4 knots | BR: Caraguatatuba 5 knots
BR: Caruaru 3 knots | BR: Caruaru 4 knots | BR: Caruaru 5 knots | BR: Cascavel 3 knots | BR: Cascavel 4 knots | BR: Cascavel 5 knots
BR: Castanhal 3 knots | BR: Castanhal 4 knots | BR: Castanhal 5 knots | BR: Catanduva 3 knots | BR: Catanduva 4 knots | BR: Catanduva 5 knots
Mortality rate per 100,000 (2010−2015)
Monthly temperature (2010−2015)
as.factor(MALE2)
FEMALES
MALES

BR (n =152) , RESPIRATORY mortality rate

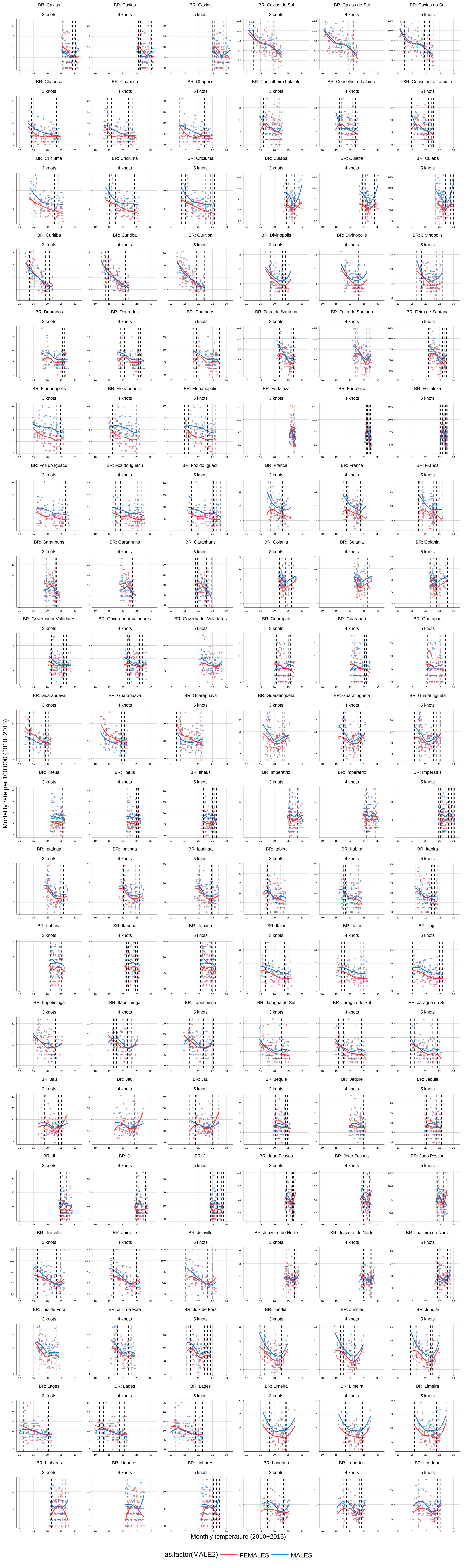

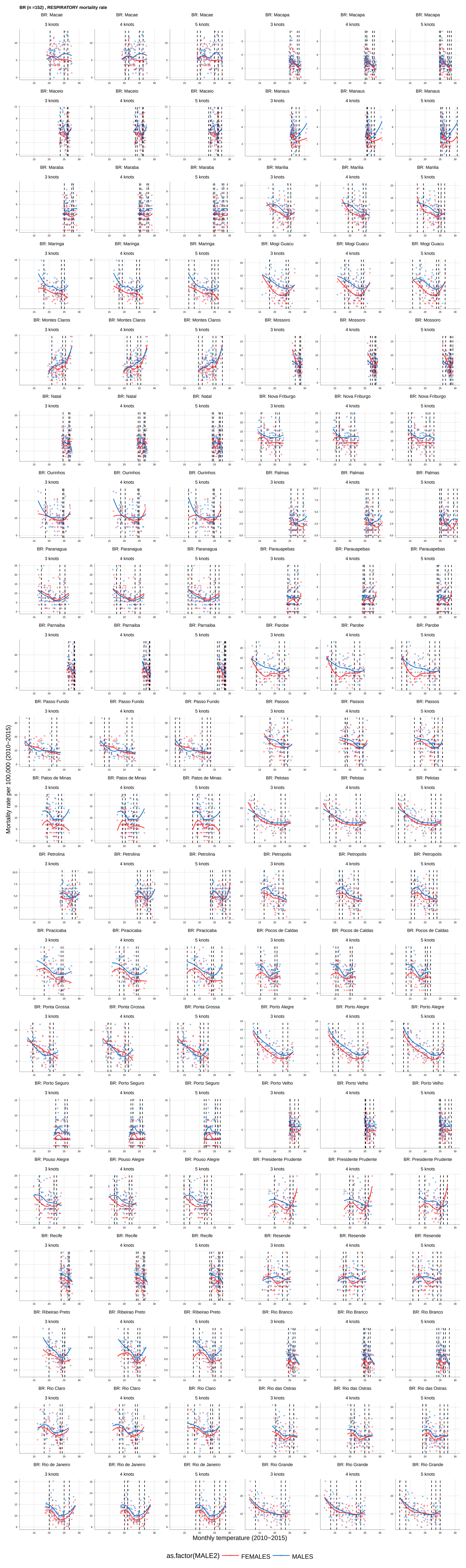
BR (n =152) , RESPIRATORY mortality rate
BR: Macae
3 knots
BR: Macae
4 knots
BR: Macae
5 knots
BR: Macapa
3 knots
BR: Macapa
4 knots
BR: Macapa
5 knots
BR: Maceio
3 knots
BR: Maceio
4 knots
BR: Maceio
5 knots
BR: Manaus
3 knots
BR: Manaus
4 knots
BR: Manaus
5 knots
BR: Maraba
3 knots
BR: Maraba
4 knots
BR: Maraba
5 knots
BR: Marilia
3 knots
BR: Marilia
4 knots
BR: Marilia
5 knots
BR: Maringa
3 knots
BR: Maringa
4 knots
BR: Maringa
5 knots
BR: Mogi Guacu
3 knots
BR: Mogi Guacu
4 knots
BR: Mogi Guacu
5 knots
BR: Montes Claros
3 knots
BR: Montes Claros
4 knots
BR: Montes Claros
5 knots
BR: Mossoro
3 knots
BR: Mossoro
4 knots
BR: Mossoro
5 knots
BR: Natal
3 knots
BR: Natal
4 knots
BR: Natal
5 knots
BR: Nova Friburgo
3 knots
BR: Nova Friburgo
4 knots
BR: Nova Friburgo
5 knots
BR: Ourinhos
3 knots
BR: Ourinhos
4 knots
BR: Ourinhos
5 knots
BR: Palmas
3 knots
BR: Palmas
4 knots
BR: Palmas
5 knots
BR: Paranagua
3 knots
BR: Paranagua
4 knots
BR: Paranagua
5 knots
BR: Parauapebas
3 knots
BR: Parauapebas
4 knots
BR: Parauapebas
5 knots
BR: Parnaiba
3 knots
BR: Parnaiba
4 knots
BR: Parnaiba
5 knots
BR: Parobe
3 knots
BR: Parobe
4 knots
BR: Parobe
5 knots
BR: Passo Fundo
3 knots
BR: Passo Fundo
4 knots
BR: Passo Fundo
5 knots
BR: Passos
3 knots
BR: Passos
4 knots
BR: Passos
5 knots
BR: Patos de Minas
3 knots
BR: Patos de Minas
4 knots
BR: Patos de Minas
5 knots
BR: Pelotas
3 knots
BR: Pelotas
4 knots
BR: Pelotas
5 knots
BR: Petrolina
3 knots
BR: Petrolina
4 knots
BR: Petrolina
5 knots
BR: Petropolis
3 knots
BR: Petropolis
4 knots
BR: Petropolis
5 knots
BR: Piracicaba
3 knots
BR: Piracicaba
4 knots
BR: Piracicaba
5 knots
BR: Pocos de Caldas
3 knots
BR: Pocos de Caldas
4 knots
BR: Pocos de Caldas
5 knots
BR: Ponta Grossa
3 knots
BR: Ponta Grossa
4 knots
BR: Ponta Grossa
5 knots
BR: Porto Alegre
3 knots
BR: Porto Alegre
4 knots
BR: Porto Alegre
5 knots
BR: Porto Seguro
3 knots
BR: Porto Seguro
4 knots
BR: Porto Seguro
5 knots
BR: Porto Velho
3 knots
BR: Porto Velho
4 knots
BR: Porto Velho
5 knots
BR: Pouso Alegre
3 knots
BR: Pouso Alegre
4 knots
BR: Pouso Alegre
5 knots
BR: Presidente Prudente
3 knots
BR: Presidente Prudente
4 knots
BR: Presidente Prudente
5 knots
BR: Recife
3 knots
BR: Recife
4 knots
BR: Recife
5 knots
BR: Resende
3 knots
BR: Resende
4 knots
BR: Resende
5 knots
BR: Ribeirao Preto
3 knots
BR: Ribeirao Preto
4 knots
BR: Ribeirao Preto
5 knots
BR: Rio Branco
3 knots
BR: Rio Branco
4 knots
BR: Rio Branco
5 knots
BR: Rio Claro
3 knots
BR: Rio Claro
4 knots
BR: Rio Claro
5 knots
BR: Rio das Ostras
3 knots
BR: Rio das Ostras
4 knots
BR: Rio das Ostras
5 knots
BR: Rio de Janeiro
3 knots
BR: Rio de Janeiro
4 knots
BR: Rio de Janeiro
5 knots
BR: Rio Grande
3 knots
BR: Rio Grande
4 knots
BR: Rio Grande
5 knots
Mortality rate per 100,000 (2010–2015)
Monthly temperature (2010–2015)
as.factor(MALE2)
FEMALES
MALES

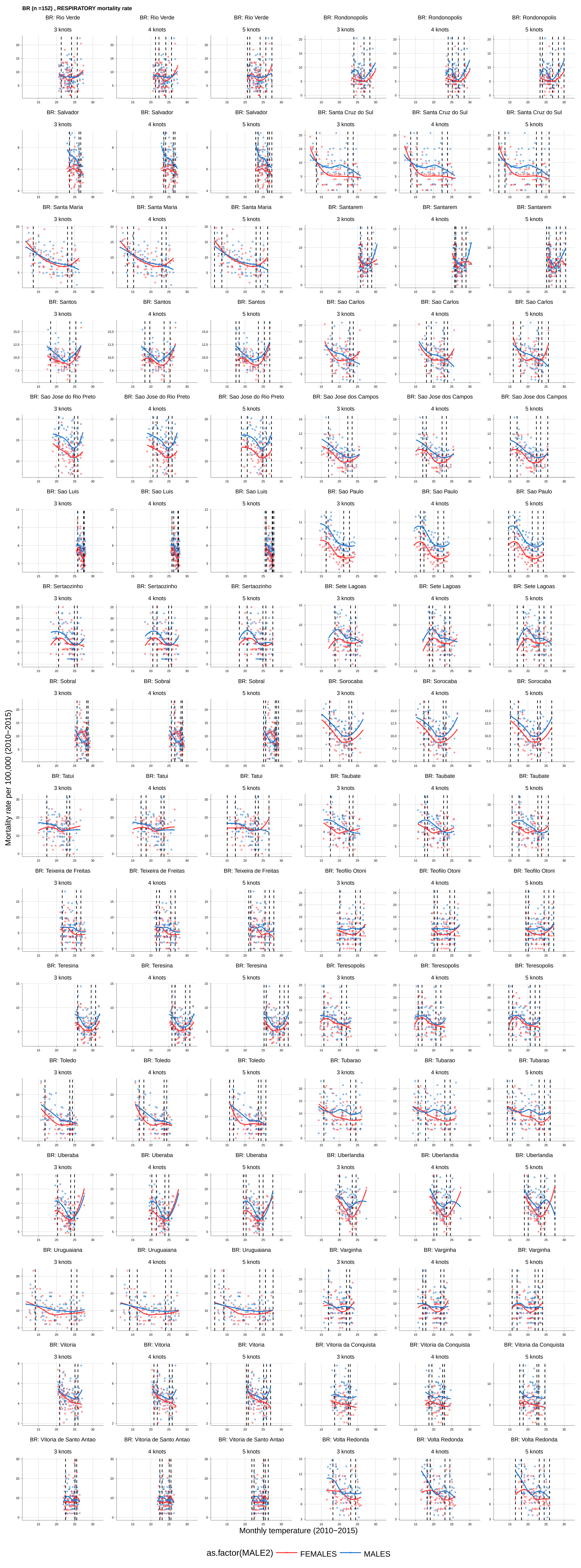
BR (n =152) , RESPIRATORY mortality rate
BR: Rio Verde
3 knots
BR: Rio Verde
4 knots
BR: Rio Verde
5 knots
BR: Rondonopolis
3 knots
BR: Rondonopolis
4 knots
BR: Rondonopolis
5 knots
BR: Salvador
3 knots
BR: Salvador
4 knots
BR: Salvador
5 knots
BR: Santa Cruz do Sul
3 knots
BR: Santa Cruz do Sul
4 knots
BR: Santa Cruz do Sul
5 knots
BR: Santa Maria
3 knots
BR: Santa Maria
4 knots
BR: Santa Maria
5 knots
BR: Santarem
3 knots
BR: Santarem
4 knots
BR: Santarem
5 knots
BR: Santos
3 knots
BR: Santos
4 knots
BR: Santos
5 knots
BR: Sao Carlos
3 knots
BR: Sao Carlos
4 knots
BR: Sao Carlos
5 knots
BR: Sao Jose do Rio Preto
3 knots
BR: Sao Jose do Rio Preto
4 knots
BR: Sao Jose do Rio Preto
5 knots
BR: Sao Jose dos Campos
3 knots
BR: Sao Jose dos Campos
4 knots
BR: Sao Jose dos Campos
5 knots
BR: Sao Luis
3 knots
BR: Sao Luis
4 knots
BR: Sao Luis
5 knots
BR: Sao Paulo
3 knots
BR: Sao Paulo
4 knots
BR: Sao Paulo
5 knots
BR: Sertaozinho
3 knots
BR: Sertaozinho
4 knots
BR: Sertaozinho
5 knots
BR: Sete Lagoas
3 knots
BR: Sete Lagoas
4 knots
BR: Sete Lagoas
5 knots
BR: Sobral
3 knots
BR: Sobral
4 knots
BR: Sobral
5 knots
BR: Sorocaba
3 knots
BR: Sorocaba
4 knots
BR: Sorocaba
5 knots
BR: Tatui
3 knots
BR: Tatui
4 knots
BR: Tatui
5 knots
BR: Taubate
3 knots
BR: Taubate
4 knots
BR: Taubate
5 knots
BR: Teixeira de Freitas
3 knots
BR: Teixeira de Freitas
4 knots
BR: Teixeira de Freitas
5 knots
BR: Teofilo Otoni
3 knots
BR: Teofilo Otoni
4 knots
BR: Teofilo Otoni
5 knots
BR: Teresina
3 knots
BR: Teresina
4 knots
BR: Teresina
5 knots
BR: Teresopolis
3 knots
BR: Teresopolis
4 knots
BR: Teresopolis
5 knots
BR: Toledo
3 knots
BR: Toledo
4 knots
BR: Toledo
5 knots
BR: Tubarao
3 knots
BR: Tubarao
4 knots
BR: Tubarao
5 knots
BR: Uberaba
3 knots
BR: Uberaba
4 knots
BR: Uberaba
5 knots
BR: Uberlandia
3 knots
BR: Uberlandia
4 knots
BR: Uberlandia
5 knots
BR: Uruguaiana
3 knots
BR: Uruguaiana
4 knots
BR: Uruguaiana
5 knots
BR: Varginha
3 knots
BR: Varginha
4 knots
BR: Varginha
5 knots
BR: Vitoria
3 knots
BR: Vitoria
4 knots
BR: Vitoria
5 knots
BR: Vitoria da Conquista
3 knots
BR: Vitoria da Conquista
4 knots
BR: Vitoria da Conquista
5 knots
BR: Vitoria de Santo Antao
3 knots
BR: Vitoria de Santo Antao
4 knots
BR: Vitoria de Santo Antao
5 knots
BR: Volta Redonda
3 knots
BR: Volta Redonda
4 knots
BR: Volta Redonda
5 knots
Mortality rate per 100,000 (2010−2015)
Monthly temperature (2010−2015)
as.factor(MALE2)
FEMALES
MALES

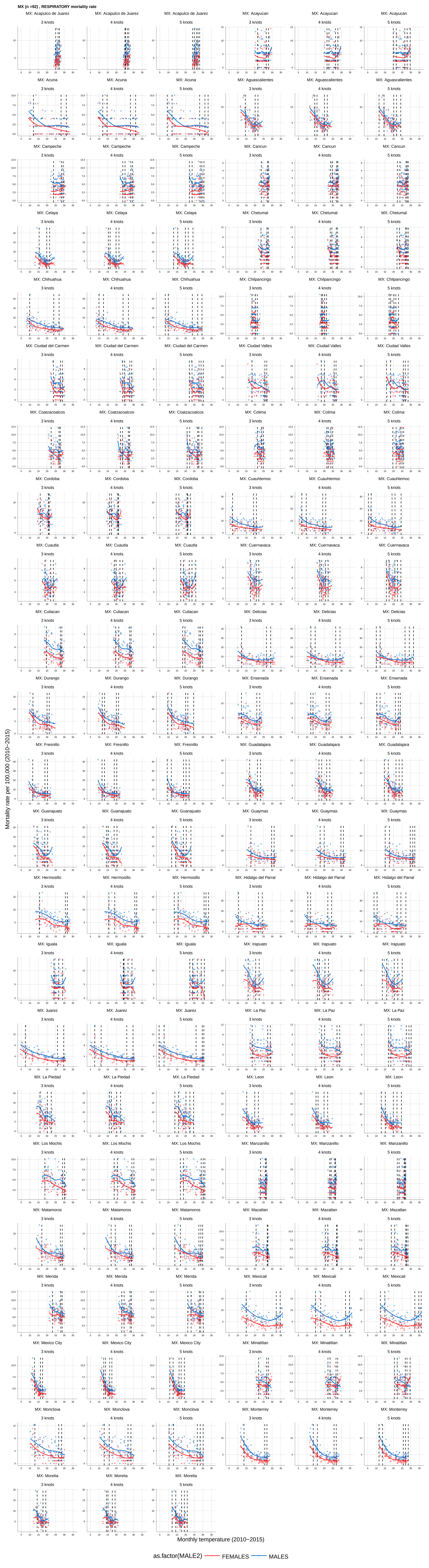
MX (n =92) , RESPIRATORY mortality rate
MX: Acapulco de Juarez
3 knots
MX: Acapulco de Juarez
4 knots
MX: Acapulco de Juarez
5 knots
MX: Acayucan
3 knots
MX: Acayucan
4 knots
MX: Acayucan
5 knots
MX: Acuna
3 knots
MX: Acuna
4 knots
MX: Acuna
5 knots
MX: Aguascalientes
3 knots
MX: Aguascalientes
4 knots
MX: Aguascalientes
5 knots
MX: Campeche
3 knots
MX: Campeche
4 knots
MX: Campeche
5 knots
MX: Cancun
3 knots
MX: Cancun
4 knots
MX: Cancun
5 knots
MX: Celaya
3 knots
MX: Celaya
4 knots
MX: Celaya
5 knots
MX: Chetumal
3 knots
MX: Chetumal
4 knots
MX: Chetumal
5 knots
MX: Chihuahua
3 knots
MX: Chihuahua
4 knots
MX: Chihuahua
5 knots
MX: Chilpancingo
3 knots
MX: Chilpancingo
4 knots
MX: Chilpancingo
5 knots
MX: Ciudad del Carmen
3 knots
MX: Ciudad del Carmen
4 knots
MX: Ciudad del Carmen
5 knots
MX: Ciudad Valles
3 knots
MX: Ciudad Valles
4 knots
MX: Ciudad Valles
5 knots
MX: Coatzacoalcos
3 knots
MX: Coatzacoalcos
4 knots
MX: Coatzacoalcos
5 knots
MX: Colima
3 knots
MX: Colima
4 knots
MX: Colima
5 knots
MX: Cordoba
3 knots
MX: Cordoba
4 knots
MX: Cordoba
5 knots
MX: Cuauhtemoc
3 knots
MX: Cuauhtemoc
4 knots
MX: Cuauhtemoc
5 knots
MX: Cuautla
3 knots
MX: Cuautla
4 knots
MX: Cuautla
5 knots
MX: Cuernavaca
3 knots
MX: Cuernavaca
4 knots
MX: Cuernavaca
5 knots
MX: Culiacan
3 knots
MX: Culiacan
4 knots
MX: Culiacan
5 knots
MX: Delicias
3 knots
MX: Delicias
4 knots
MX: Delicias
5 knots
MX: Durango
3 knots
MX: Durango
4 knots
MX: Durango
5 knots
MX: Ensenada
3 knots
MX: Ensenada
4 knots
MX: Ensenada
5 knots
MX: Fresnillo
3 knots
MX: Fresnillo
4 knots
MX: Fresnillo
5 knots
MX: Guadalajara
3 knots
MX: Guadalajara
4 knots
MX: Guadalajara
5 knots
MX: Guanajuato
3 knots
MX: Guanajuato
4 knots
MX: Guanajuato
5 knots
MX: Guaymas
3 knots
MX: Guaymas
4 knots
MX: Guaymas
5 knots
MX: Hermosillo
3 knots
MX: Hermosillo
4 knots
MX: Hermosillo
5 knots
MX: Hidalgo del Parral
3 knots
MX: Hidalgo del Parral
4 knots
MX: Hidalgo del Parral
5 knots
MX: Iguala
3 knots
MX: Iguala
4 knots
MX: Iguala
5 knots
MX: Irapuato
3 knots
MX: Irapuato
4 knots
MX: Irapuato
5 knots
MX: Juarez
3 knots
MX: Juarez
4 knots
MX: Juarez
5 knots
MX: La Paz
3 knots
MX: La Paz
4 knots
MX: La Paz
5 knots
MX: La Piedad
3 knots
MX: La Piedad
4 knots
MX: La Piedad
5 knots
MX: Leon
3 knots
MX: Leon
4 knots
MX: Leon
5 knots
MX: Los Mochis
3 knots
MX: Los Mochis
4 knots
MX: Los Mochis
5 knots
MX: Manzanillo
3 knots
MX: Manzanillo
4 knots
MX: Manzanillo
5 knots
MX: Matamoros
3 knots
MX: Matamoros
4 knots
MX: Matamoros
5 knots
MX: Mazatlan
3 knots
MX: Mazatlan
4 knots
MX: Mazatlan
5 knots
MX: Merida
3 knots
MX: Merida
4 knots
MX: Merida
5 knots
MX: Mexicali
3 knots
MX: Mexicali
4 knots
MX: Mexicali
5 knots
MX: Mexico City
3 knots
MX: Mexico City
4 knots
MX: Mexico City
5 knots
MX: Minatitlan
3 knots
MX: Minatitlan
4 knots
MX: Minatitlan
5 knots
MX: Monclova
3 knots
MX: Monclova
4 knots
MX: Monclova
5 knots
MX: Monterrey
3 knots
MX: Monterrey
4 knots
MX: Monterrey
5 knots
MX: Morelia
3 knots
MX: Morelia
4 knots
MX: Morelia
5 knots
Mortality rate per 100,000 (2010−2015)
Monthly temperature (2010−2015)
as.factor(MALE2)
FEMALES
MALES

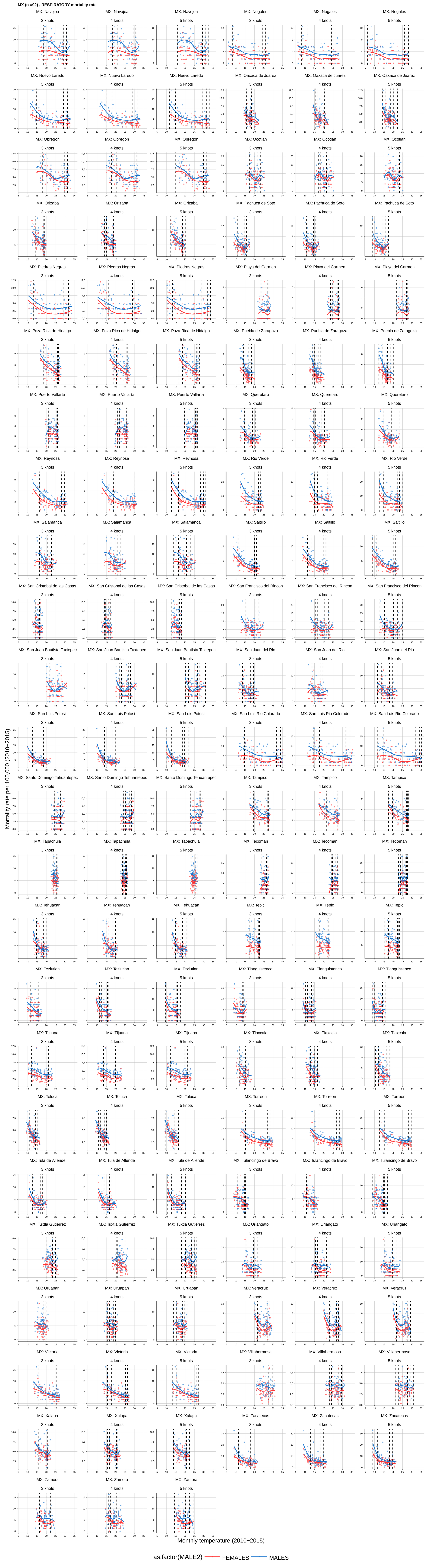
MX (n =92) , RESPIRATORY mortality rate
MX: Navojoa
MX: Nogales
MX: Nuevo Laredo
MX: Oaxaca de Juarez
MX: Obregon
MX: Ocotlan
MX: Orizaba
MX: Pachuca de Soto
MX: Piedras Negras
MX: Playa del Carmen
MX: Poza Rica de Hidalgo
MX: Puebla de Zaragoza
MX: Puerto Vallarta
MX: Queretaro
MX: Reynosa
MX: Rio Verde
MX: Salamanca
MX: Saltillo
MX: San Cristobal de las Casas
MX: San Francisco del Rincon
MX: San Juan Bautista Tuxtepec
MX: San Juan del Rio
MX: San Luis Potosi
MX: San Luis Rio Colorado
MX: Santo Domingo Tehuantepec
MX: Tampico
MX: Tapachula
MX: Tecoman
MX: Tehuacan
MX: Tepic
MX: Teziutlan
MX: Tianguistenco
MX: Tijuana
MX: Tlaxcala
MX: Toluca
MX: Torreon
MX: Tula de Allende
MX: Tulancingo de Bravo
MX: Tuxtla Gutierrez
MX: Uriangato
MX: Uruapan
MX: Veracruz
MX: Victoria
MX: Villahermosa
MX: Xalapa
MX: Zacatecas
MX: Zamora
3 knots
4 knots
5 knots
Mortality rate per 100,000 (2010−2015)
Monthly temperature (2010−2015)
as.factor(MALE2)
FEMALES
MALES

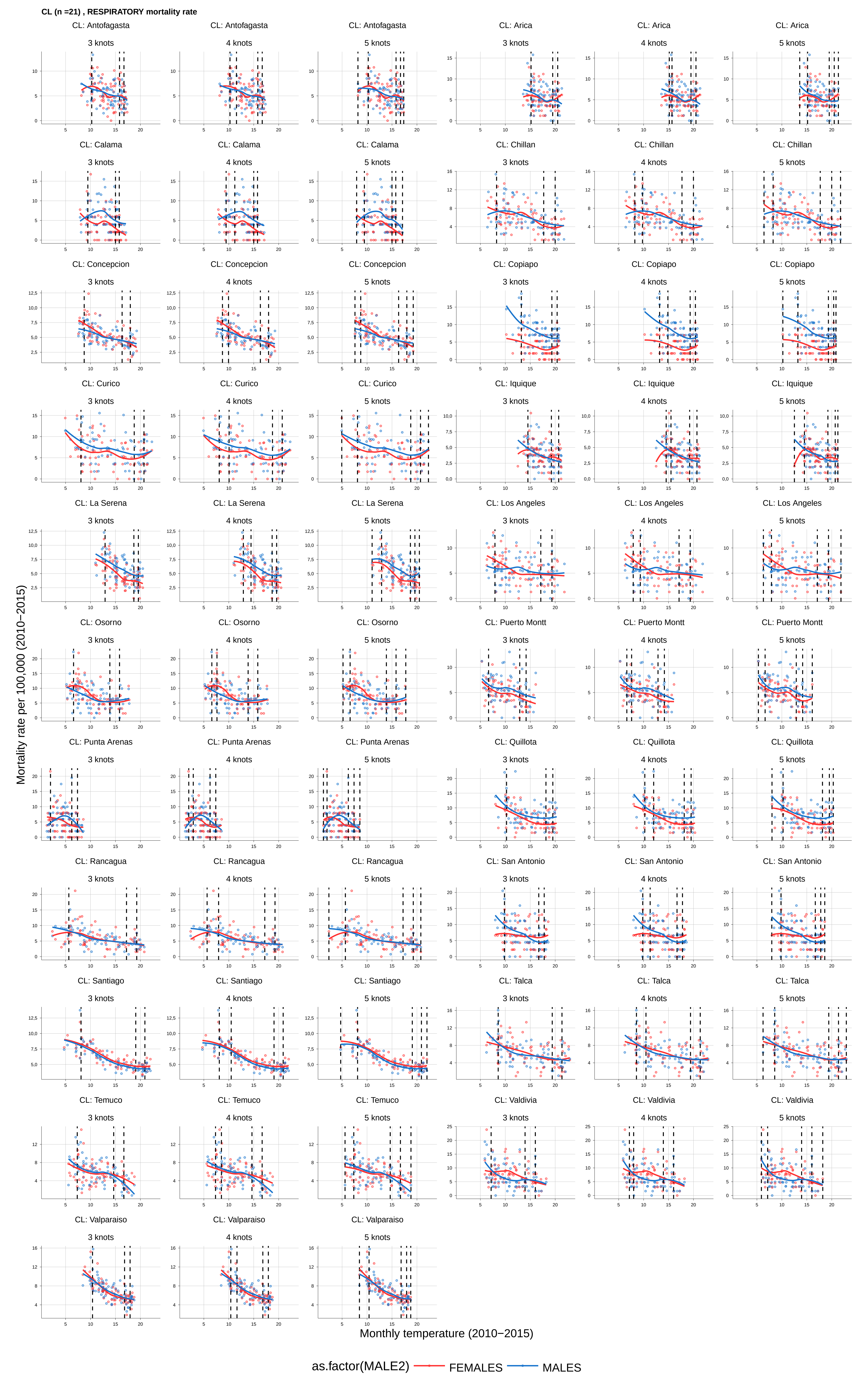
CL (n =21) , RESPIRATORY mortality rate
3 knots
4 knots
5 knots
Mortality rate per 100,000 (2010−2015)
Monthly temperature (2010−2015)
as.factor(MALE2)
FEMALES
MALES

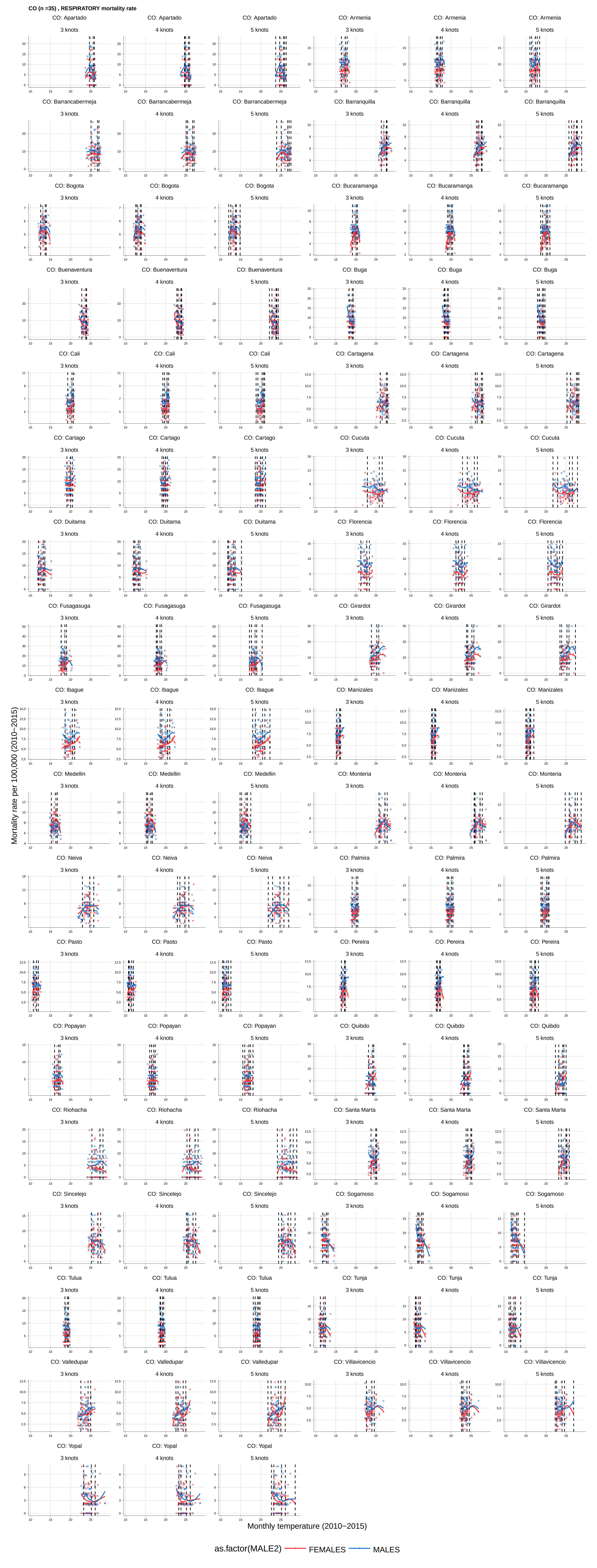
CO (n =35) , RESPIRATORY mortality rate
3 knots
4 knots
5 knots
Mortality rate per 100,000 (2010−2015)
Monthly temperature (2010−2015)
as.factor(MALE2)
FEMALES
MALES

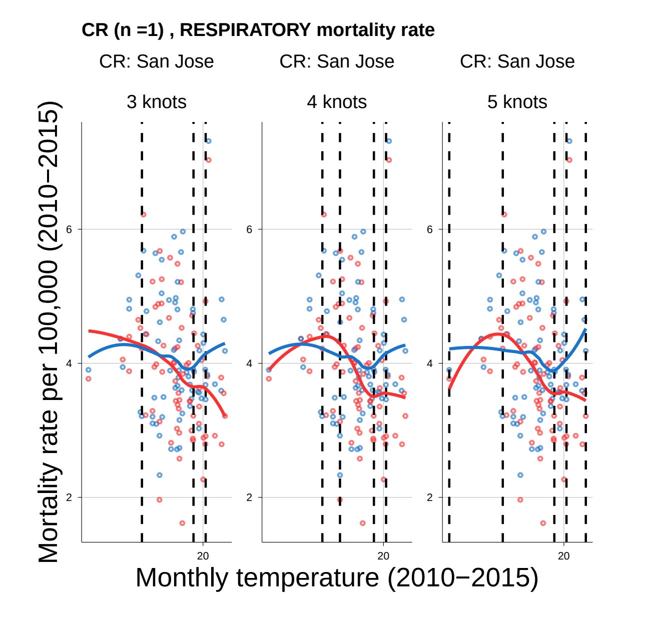
CR (n =1) , RESPIRATORY mortality rate
CR: San Jose
3 knots
4 knots
5 knots
Mortality rate per 100,000 (2010−2015)
Monthly temperature (2010−2015)

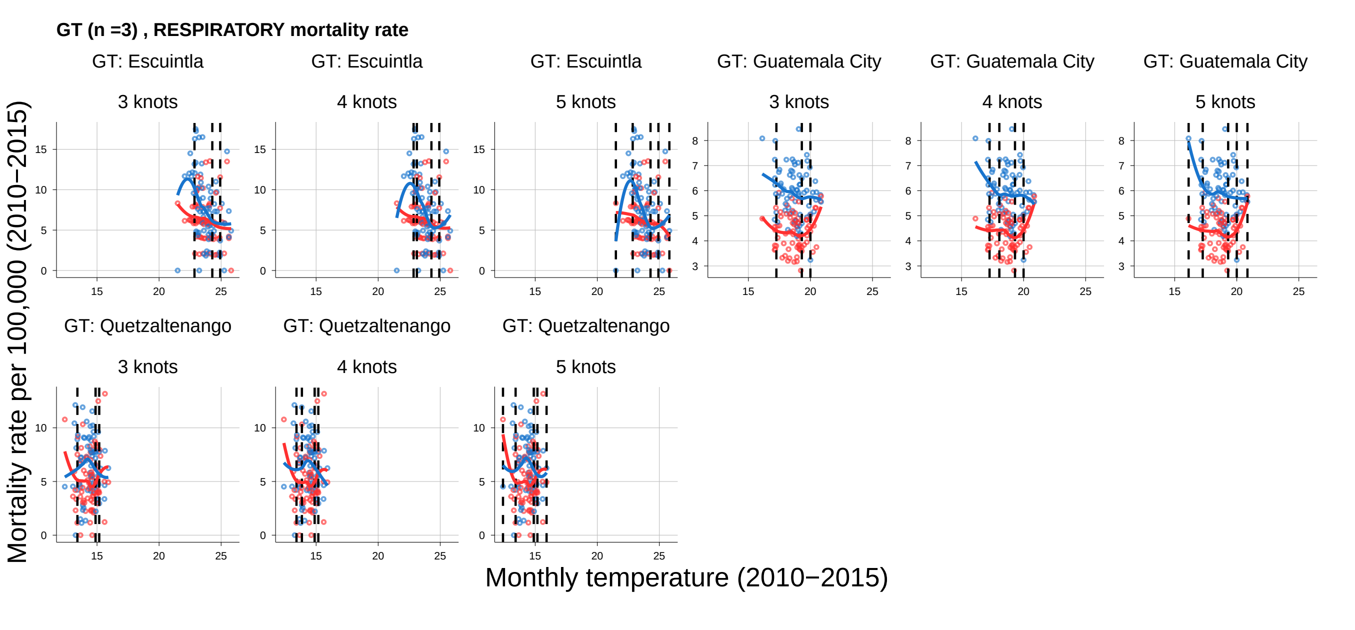
GT (n =3) , RESPIRATORY mortality rate
GT: Escuintla
GT: Guatemala City
GT: Quetzaltenango
3 knots
4 knots
5 knots
Mortality rate per 100,000 (2010−2015)
Monthly temperature (2010−2015)

PA (n =3) , RESPIRATORY mortality rate

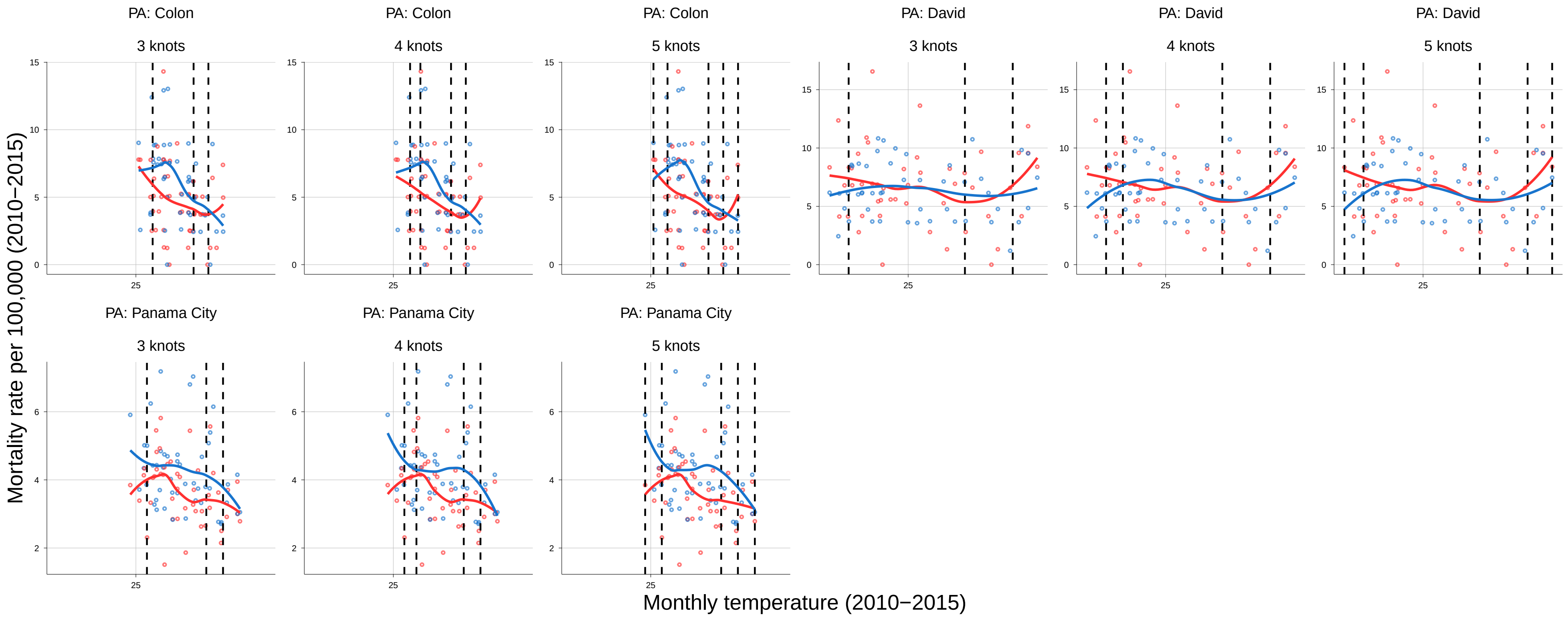

SV (n =3) , RESPIRATORY mortality rate

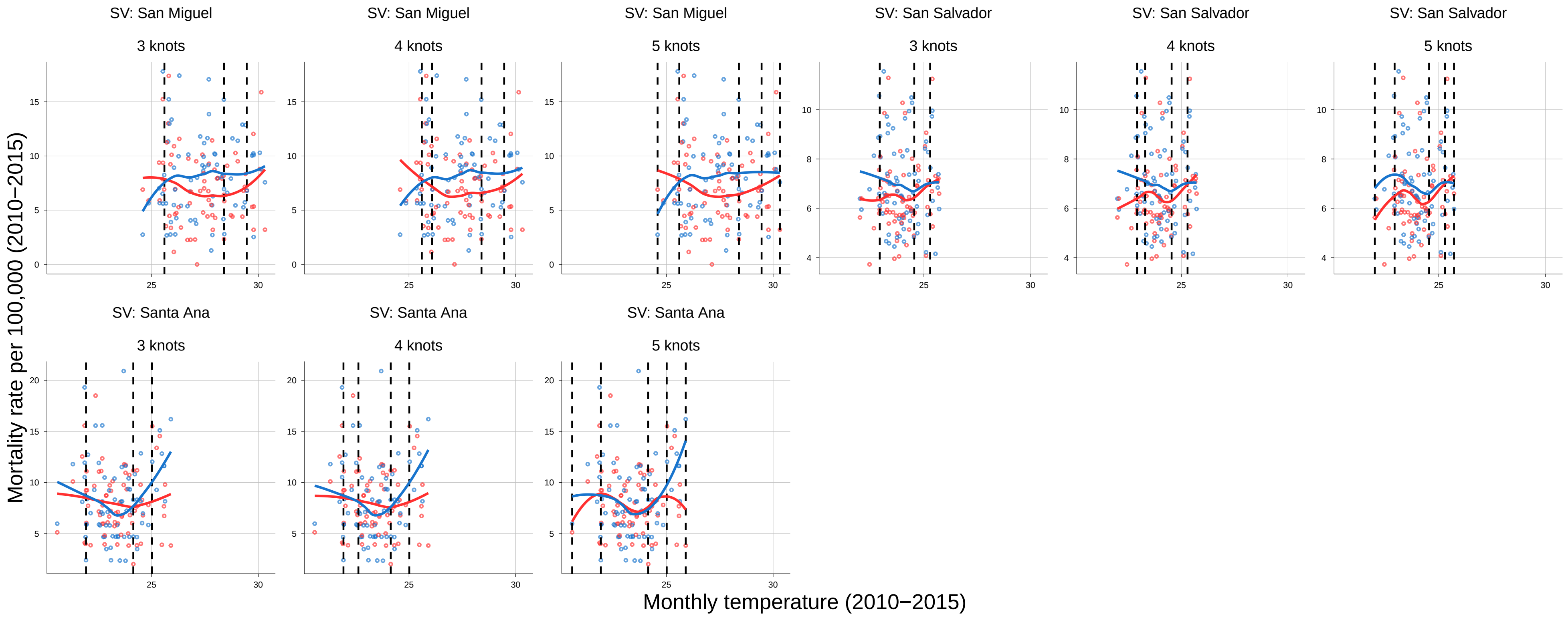

Supplement: Supplementary file 2 — Supplementary material 2 [file mmc2.pdf]
