# Supplementary figures and images for: Short-term associations between fine particulate air pollution and cardiovascular and respiratory mortality in 337 cities in Latin America
Source: Sci Total Environ. 2024 Apr 10;920:171073. doi: 10.1016/j.scitotenv.2024.171073 (PMC10918459; doi:10.1016/j.scitotenv.2024.171073)

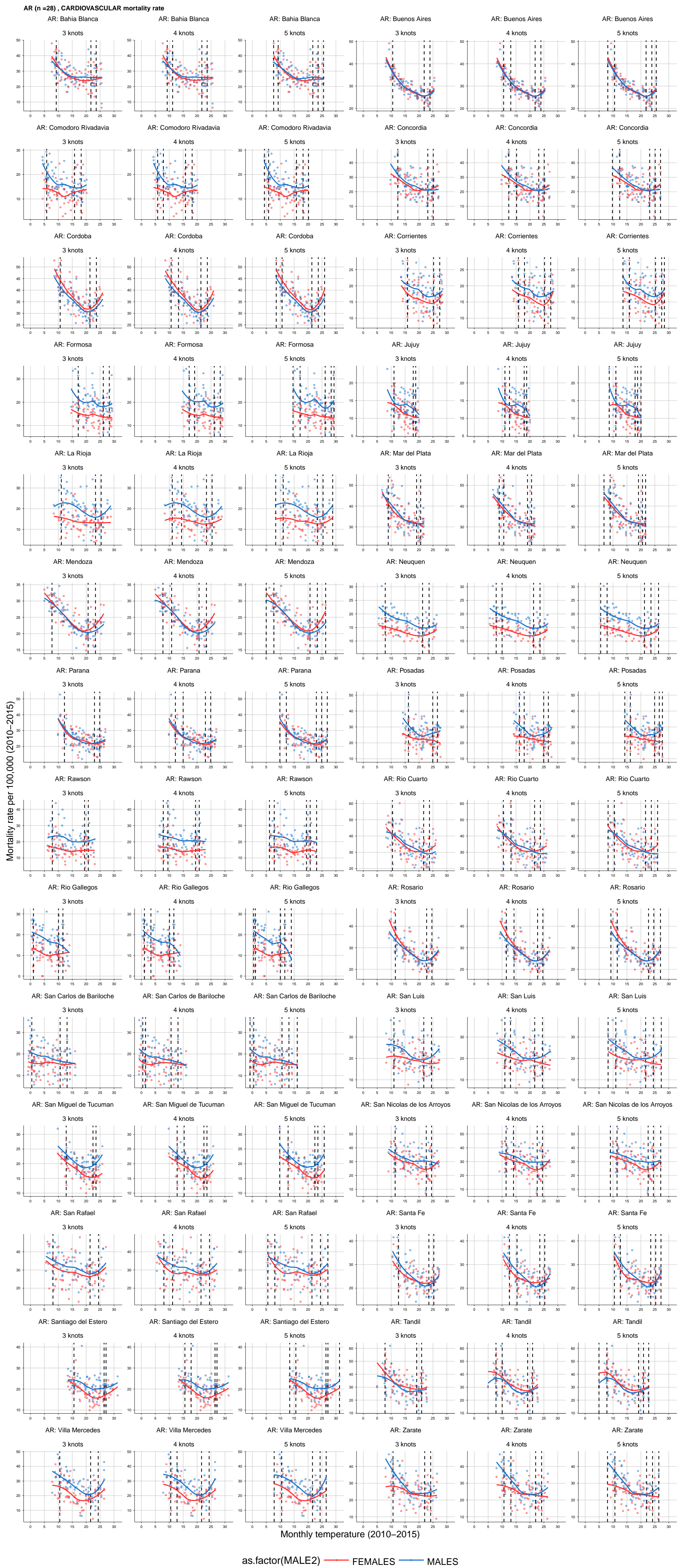

Mortality rate per 100,000 (2010–2015)

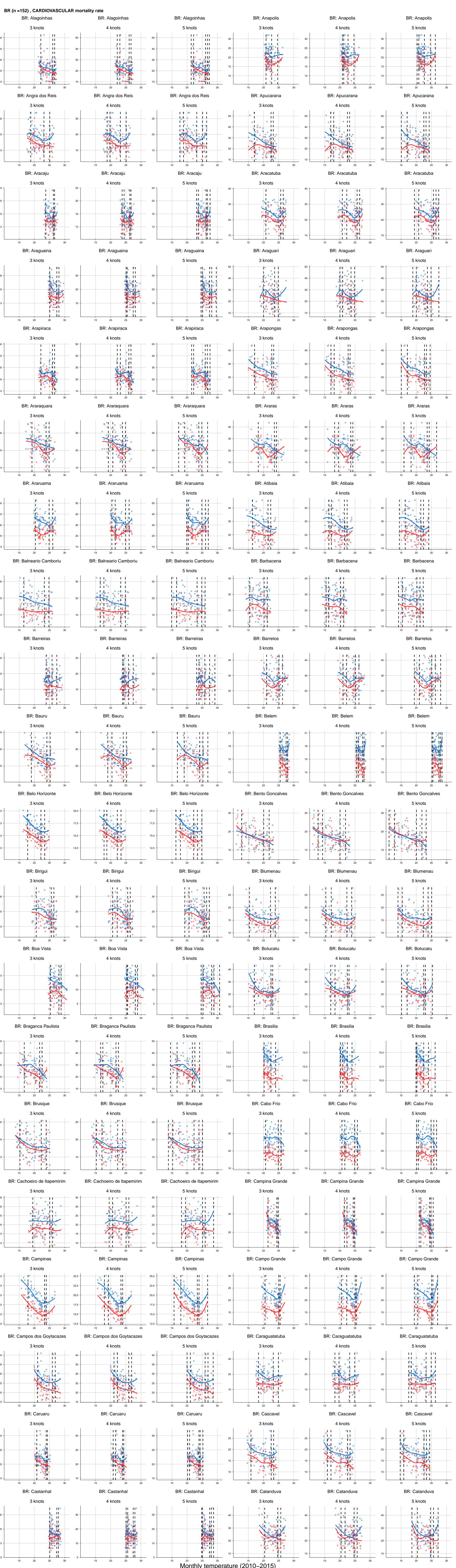

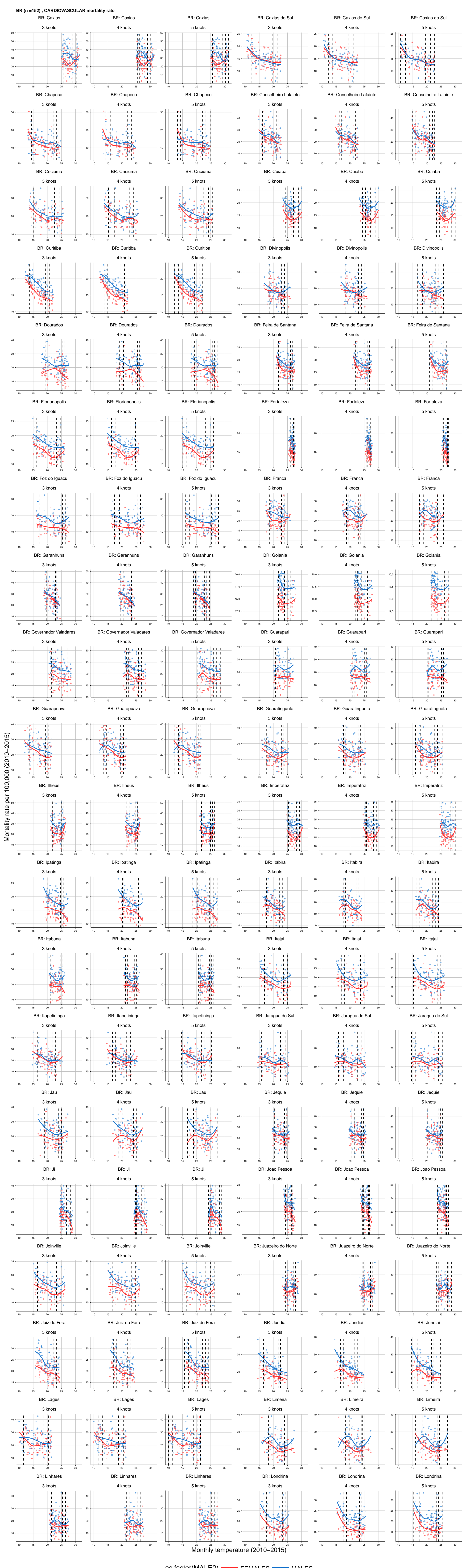

BB (n = 152) CARDIOVASCULAR mortality rate

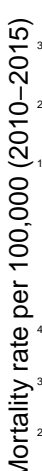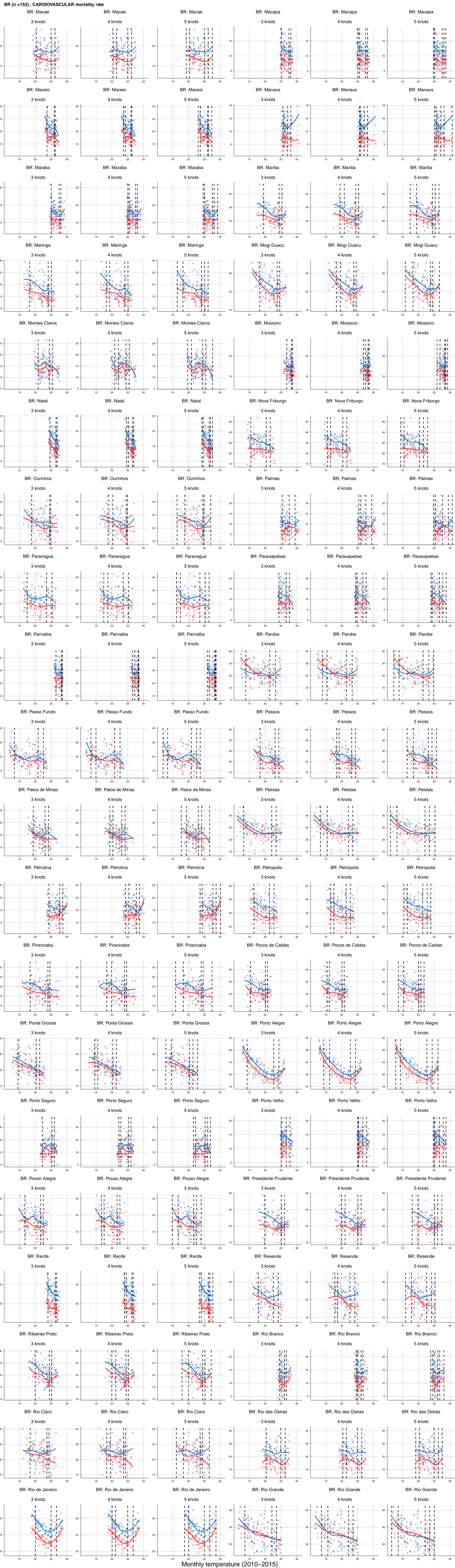

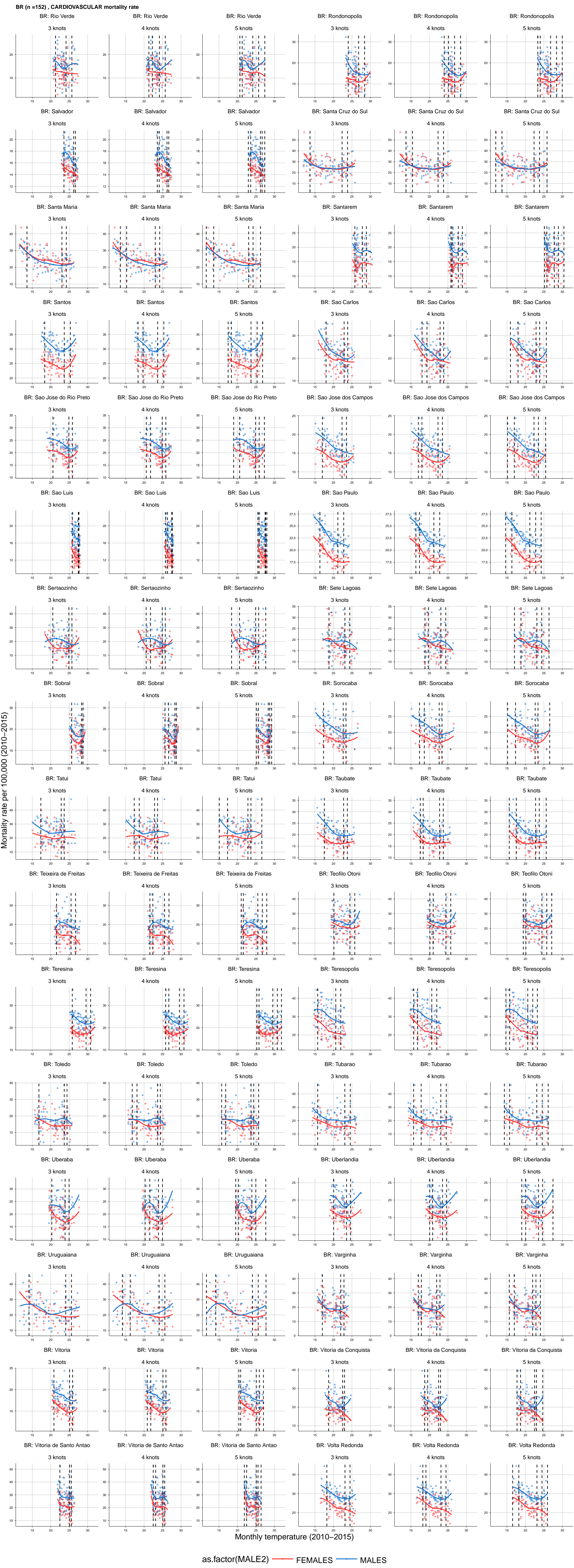

MX (n=92) , CARDIOVASCULAR mortality rate

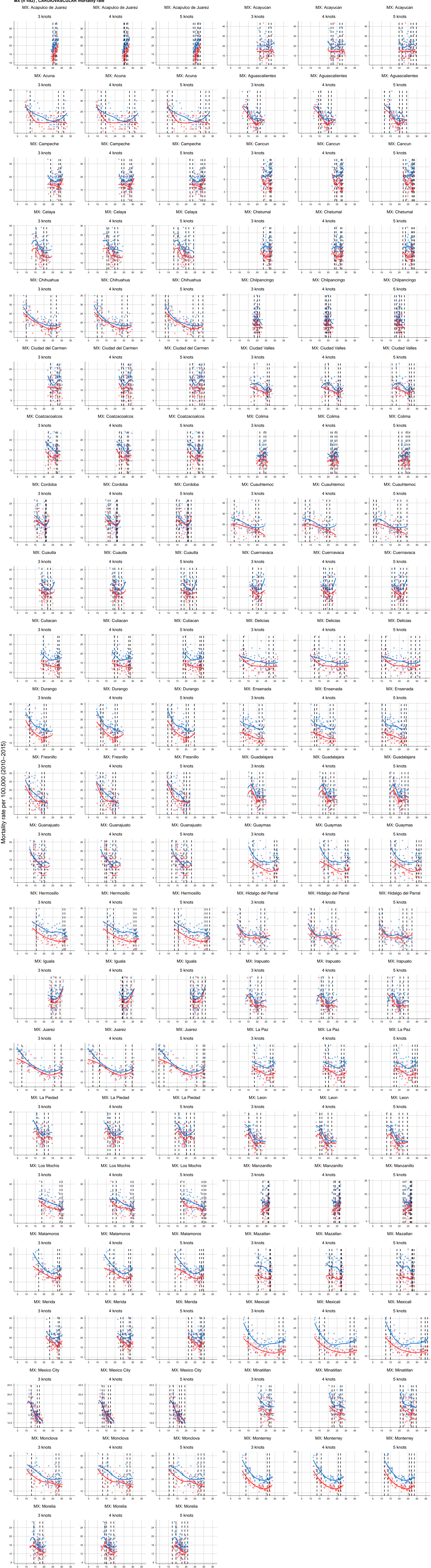

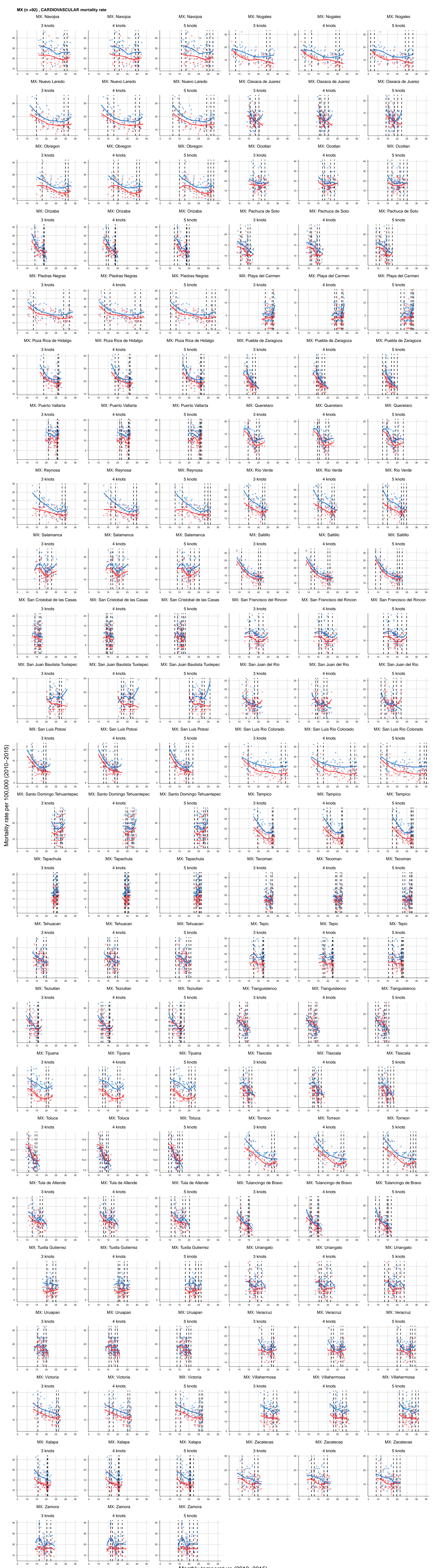

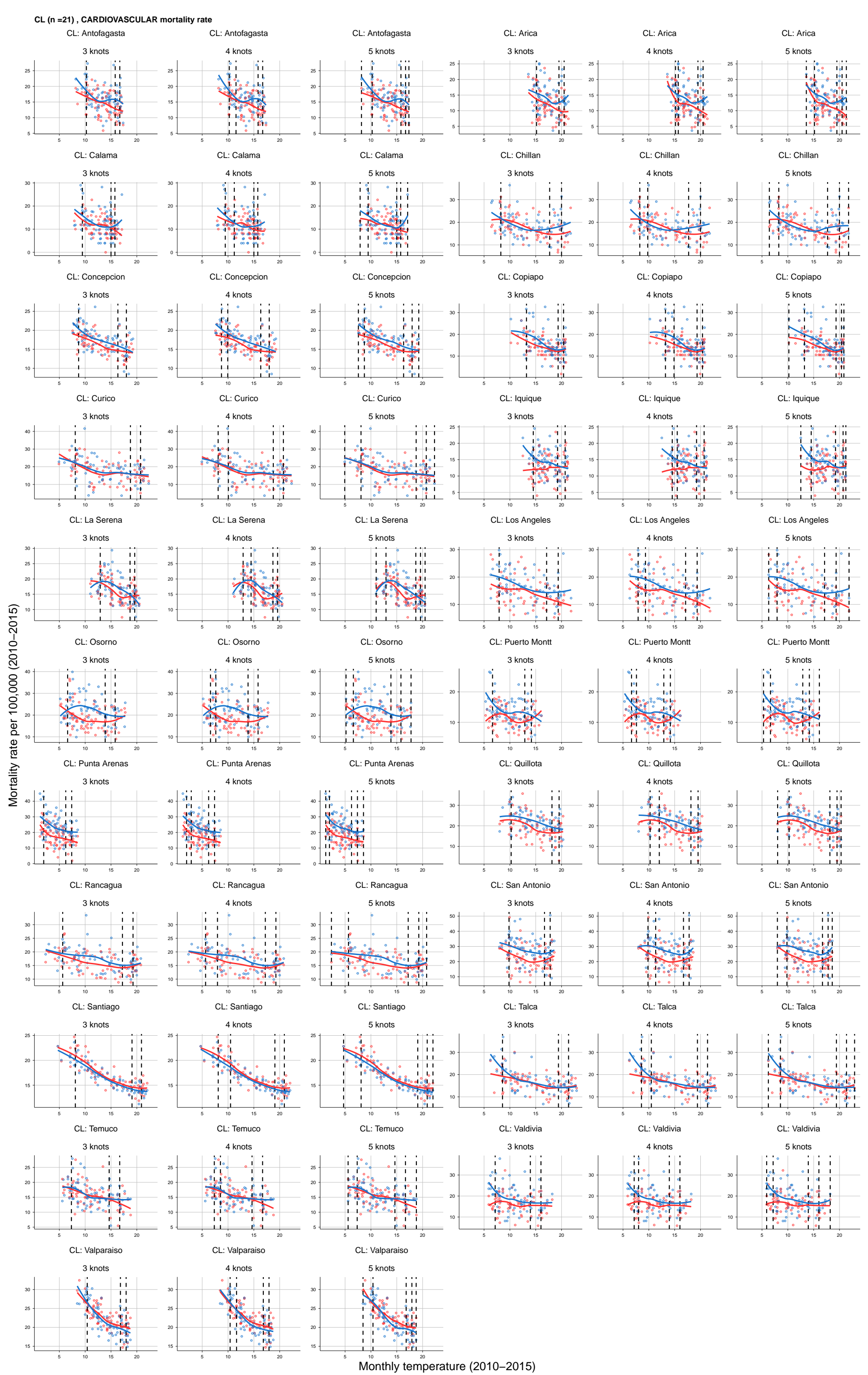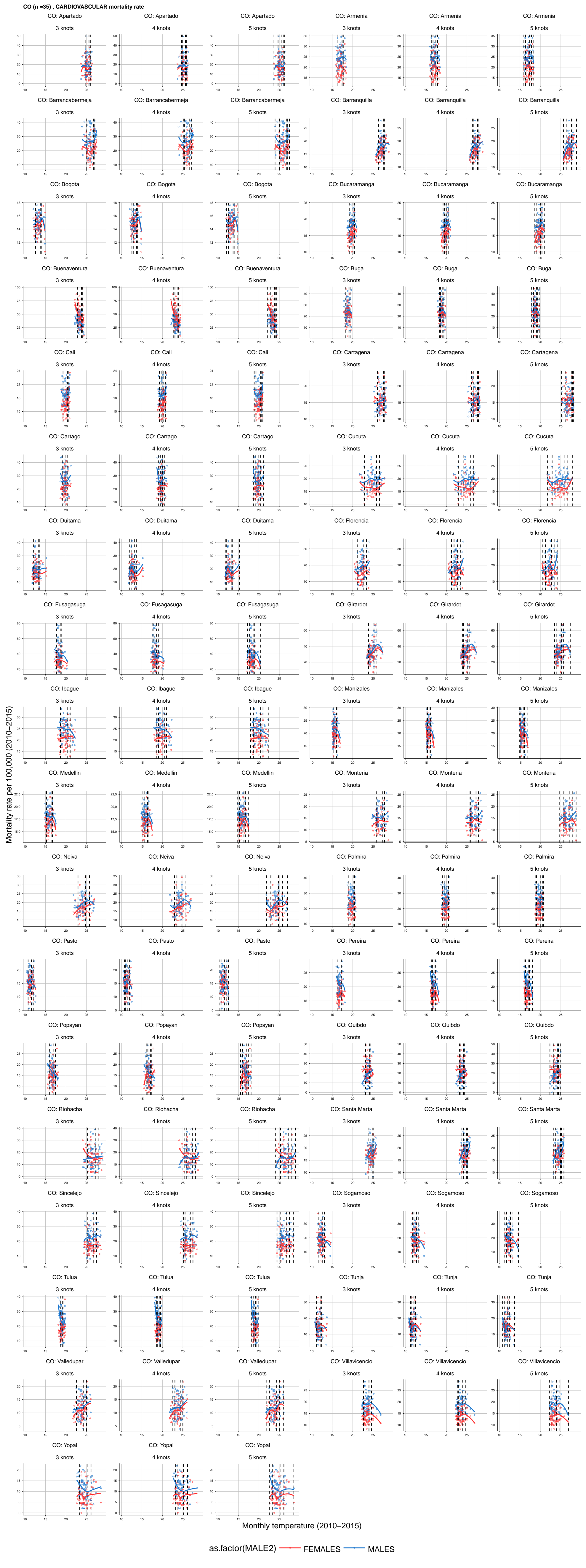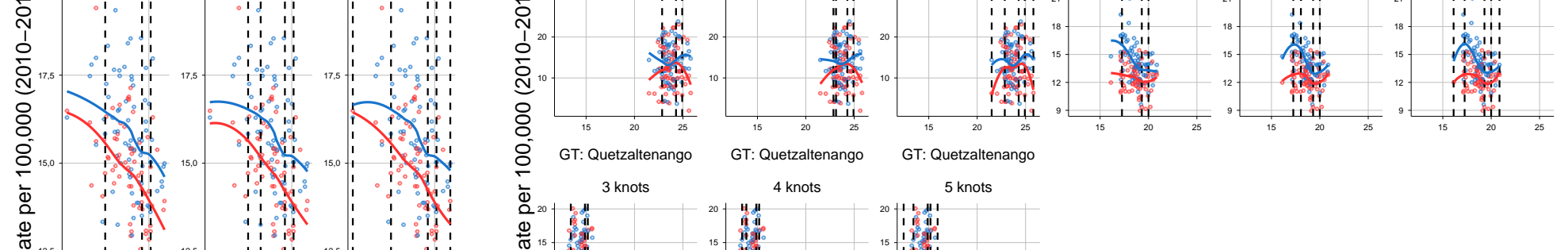

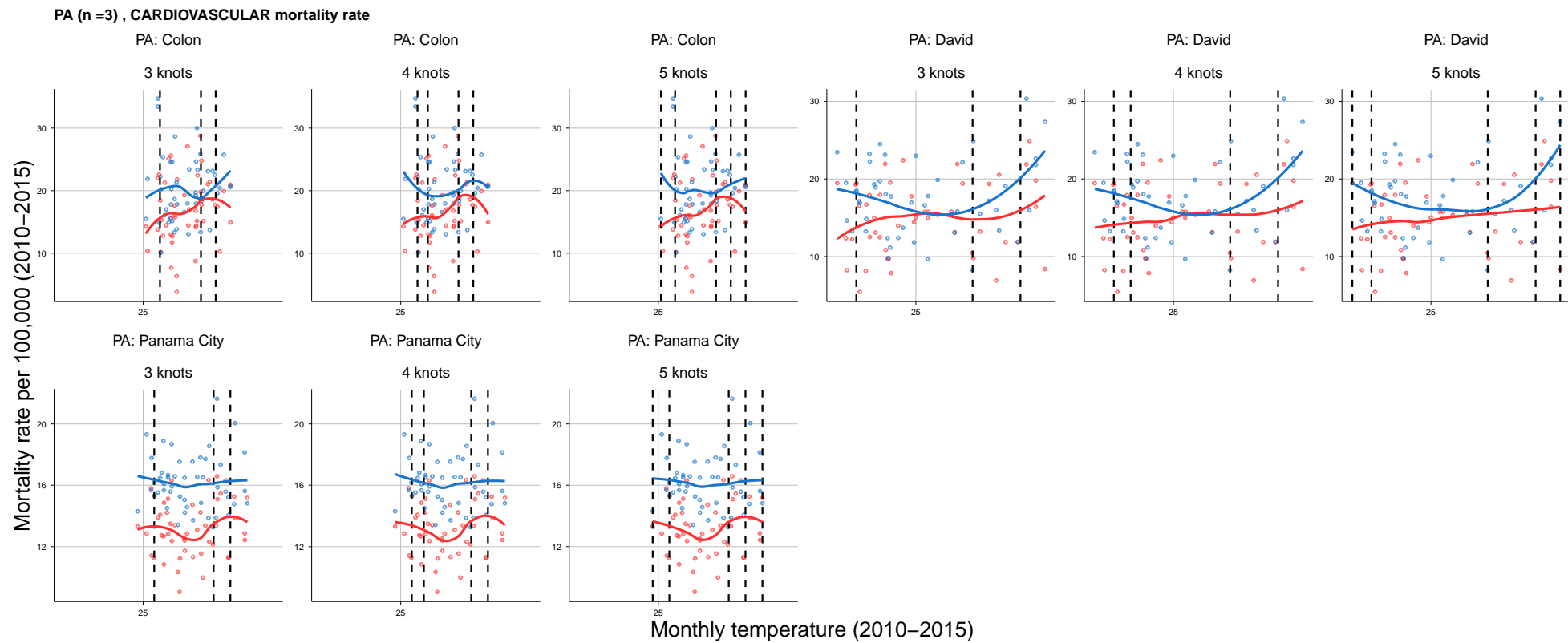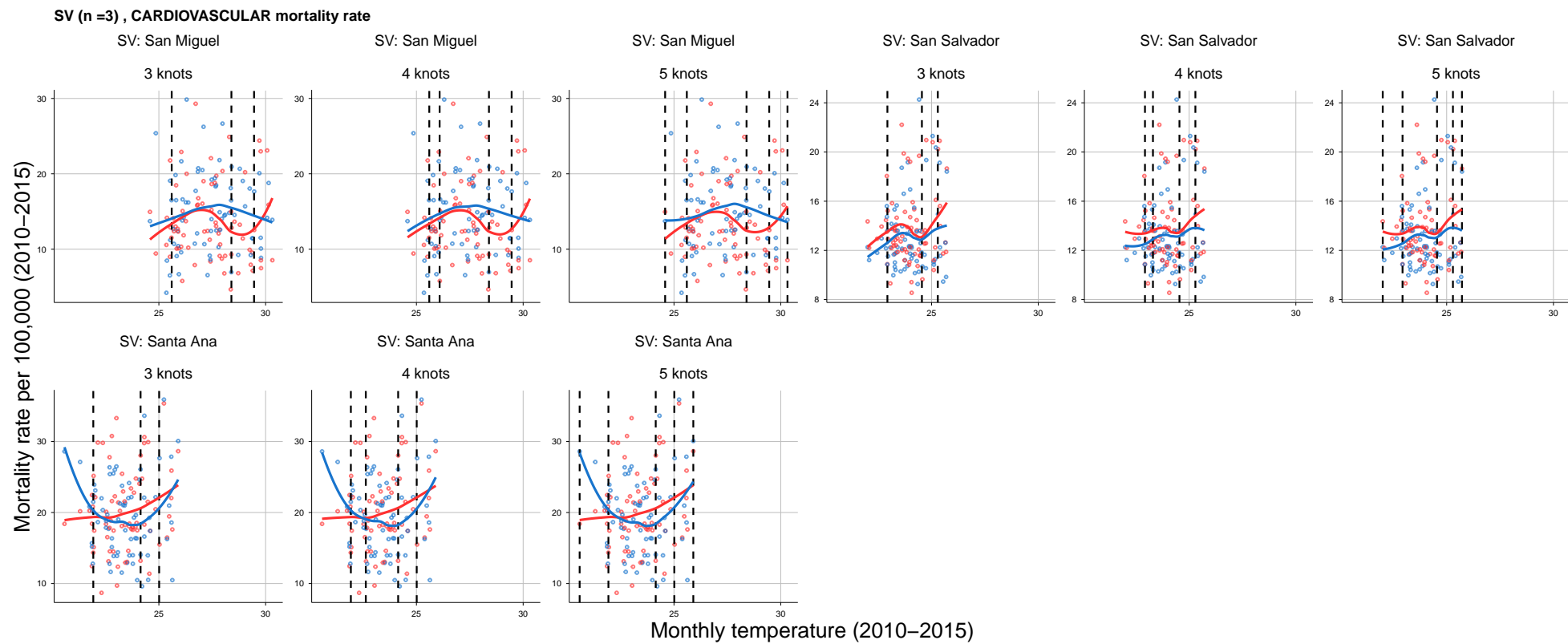

Supplement: Supplementary file 3 — Supplementary material 3 [file mmc3.pdf]
